# GDF10 attenuates MASH progression by restoring quiescent hepatic stellate cells via competitive inhibition of TGF-β/SMAD2 signaling

**DOI:** 10.7150/ijbs.123784

**Published:** 2025-10-27

**Authors:** Yajie Peng, Hongyan Lei, Jiahui Zhao, Huajuan Wang, Zheng Luo, Dixin Wang, Shujun Shi, Tianyi Wang, Jin Li, Zhiqing Pang, Bo Wang, Xuelian Xiong

**Affiliations:** 1 Ministry of Education Key Laboratory of Metabolism and Molecular Medicine, Department of Endocrinology and Metabolism, Zhongshan Hospital, Fudan University, 200020, Shanghai, China.; 2 State Key Laboratory of Genetic Engineering, School of Life Sciences, Fudan University, 200438, Shanghai, China.; 3 Key Laboratory of Smart Drug Delivery, Ministry of Education, School of Pharmacy, Fudan University, 201203, Shanghai, China.; 4 Department of Endocrinology, Xinhua Hospital Affiliated to Shanghai Jiao Tong University School of Medicine, 200092, Shanghai, China.

**Keywords:** GDF10, MASH, HSCs, liver fibrosis

## Abstract

Liver fibrosis has emerged as the primary determinant of outcomes in metabolic dysfunction-associated steatohepatitis (MASH). Quiescent hepatic stellate cells (HSCs) differentiate into activated HSCs or myofibroblasts, which drives liver fibrosis and contribute to the progressive loss of hepatic function. MASH with progressive fibrosis lacks effective therapies due to incomplete understanding of HSCs regulation. Here, we identify growth differentiation factor 10 (GDF10) as a master regulator of HSCs quiescence that ameliorates fibrosis through shifting HSC functions to restore HSC balance of transcriptional and metabolic reprogramming. Single-cell RNA sequencing revealed HSC-specific *Gdf10* expression inversely correlated with fibrotic activation. In murine models of diet-induced MASH and CCl4-induced fibrosis, AAV-mediated *Gdf10* overexpression reduced collagen deposition, serum ALT/AST, and fibrogenic gene expression without perturbing glucose or lipid metabolism. Mechanistically, GDF10 competitively bound TGF-β receptor 2 (TβR2), inhibiting SMAD2/3 phosphorylation and nuclear translocation, ultimately suppressing TGFβ1-driven extracellular matrix production, and reversing the activated HSCs phenotype and their hypermetabolic states. Leveraging this pathway, we developed liver-targeted lipid nanoparticles (LNPs) encapsulating *mGdf10* mRNA, which selectively delivered *Gdf10* to HSCs, reversed fibrosis in multiple animal models. Clinically, *GDF10* expression correlated with fibrosis severity in human cirrhotic livers. Our findings establish GDF10 as a dual-function modulator of TGF-β signaling and HSC metabolism, offering a targeted therapeutic strategy for liver fibrosis.

## Background

Liver fibrosis, a hallmark of chronic liver disease, represents a significant global health burden, particularly in the context of metabolic dysfunction-associated steatohepatitis (MASH) 1-3. As the most advanced form of metabolic dysfunction-associated steatotic liver disease (MASLD), MASH is characterized by hepatic steatosis, inflammation, and progressive fibrosis, which collectively drive the transition to cirrhosis and hepatocellular carcinoma 4, 5. The prevalence of MASLD and MASH has reached epidemic proportions, affecting over one-third of the global adult population, with fibrosis stage being the strongest predictor of liver-related mortality 6, 7. Despite its clinical significance, no approved therapies specifically target liver fibrosis, underscoring the urgent need for mechanistic insights and novel therapeutic strategies 8. The initiation of liver fibrosis is a process involving the collaboration of multiple cell types. Among these, the activation of immune cells and the dysfunction of liver sinusoidal endothelial cells (LSECs) are key initiating events that disrupt the homeostasis of the intrahepatic environment. Hepatic stellate cells (HSCs) serve as the core executors of the fibrotic process; upon receiving external activation signals, HSCs are activated and transdifferentiate into myofibroblasts, which subsequently secrete excessive amounts of extracellular matrix (ECM) components. This process ultimately leads to abnormal excessive extracellular matrix (ECM) deposition, progressive fibrosis, and the disruption of normal liver architecture 9-11. Understanding the molecular mechanisms governing HSC activation and reversion to quiescence is thus critical for developing effective antifibrotic treatments.

The activation of HSCs is orchestrated by a complex interplay of signaling pathways, with transforming growth factor-β (TGF-β) emerging as a master regulator of fibrogenesis 12, 13. TGFβ1, the most potent profibrogenic cytokine, binds to TGF-β receptor 2 (TβR2), triggering SMAD2/3 phosphorylation and nuclear translocation, which in turn upregulates ECM production and inhibits matrix degradation 14, 15. While the mechanisms driving HSC activation are well-studied, the pathways that promote HSC quiescence or inactivation remain less understood. Recent studies have highlighted the remarkable plasticity of HSCs, demonstrating that activated HSCs can revert to a quiescent-like state during fibrosis regression, a process accompanied by metabolic reprogramming and downregulation of fibrogenic genes 16, 17. This plasticity suggests that targeting HSC reversion, rather than solely inhibiting activation, could offer a promising therapeutic avenue. However, the endogenous factors that orchestrate this reversion and their interplay with metabolic and fibrogenic pathways remain elusive, limiting the development of targeted therapies.

Growth differentiation factor 10 (GDF10), a lesser-studied member of the TGF-β superfamily, has recently garnered attention for its roles in tissue repair and metabolic regulation 18, 19. Unlike other TGF-β family members, GDF10 signals through TβR2 but exhibits context-dependent antagonistic effects on TGF-β signaling, as seen in its anti-adipogenic and neuroprotective actions 19, 20. In the liver, GDF10 expression is enriched in HSCs, yet its functional significance in liver fibrosis and HSC biology has not been explored. Preliminary evidence suggests that GDF10 may modulate cellular metabolism, a key determinant of HSC activation, by suppressing glycolytic flux-a metabolic adaptation critical for myofibroblast persistence 21, 22. Given the dual role of TGF-β signaling and metabolic reprogramming in fibrosis, we hypothesized that GDF10 could serve as a natural brake on HSC activation by competitively inhibiting TGF-β/SMAD2 signaling while restoring quiescence-associated metabolic homeostasis. Unraveling this mechanism could unlock new therapeutic strategies that simultaneously target fibrogenic signaling and metabolic dysregulation in HSCs.

In this study, we identify GDF10 as a pivotal regulator of HSC quiescence and fibrosis resolution. Using single-cell RNA sequencing, we demonstrate that *Gdf10* expression is restricted to HSCs and inversely correlates with fibrotic activation across animal models and human cirrhosis. Through gain- and loss-of-function experiments, we show that *Gdf10* overexpression attenuates liver fibrosis by competitively binding TβR2, blunting SMAD2/3 phosphorylation, and suppressing TGFβ1-driven ECM deposition. Mechanistically, GDF10 reprograms HSC metabolism, reversing the glycolytic switch characteristic of activated HSCs and restoring a quiescent phenotype. Capitalizing on these insights, we developed liver-targeted lipid nanoparticles (LNPs) encapsulating *Gdf10* mRNA, which selectively deliver *Gdf10* to HSCs and reverse fibrosis in diet- and toxin-induced models without perturbing systemic metabolism. Our findings posited GDF10 as a dual-functional modulator of TGF-β signaling and HSC metabolic plasticity, offering a precision therapeutic strategy for liver fibrosis. This work not only advances our understanding of HSC biology but also provides a translatable platform for RNA-based antifibrotic therapy.

## Methods

### Animal studies

All animal studies were performed following procedures approved by the Institutional Animal Care & Use Committee at the Fudan University. Mice were housed in pathogen-free facilities under 12 h light-dark cycles with free access to water and fed with a normal chow diet (NCD). To induce MASH, C57BL/6J mice were fed with High-fat/High-fructose/High-cholesterol diet (HFFC diet, Research Diets, D09100310, 40% kcal fat, 20% kcal fructose and 2% cholesterol) for 5 months and High-fat and Methionine/Choline-deficient diet (HFMCD diet, Research Diets, A06071309, 45% kcal fat with 0.1% Methionine and no added choline) for 10 weeks respectively. In diet induced MAFLD, mice were fed with High fat diet (HFD diet, Research Diets, D12492) as indicated time. To establish injury induced liver fibrosis, CCl4 was liquified in 1:6 ratio in olive oil and was injected at 0.8ml/kg body weight intraperitoneally into mice twice per week for 3 weeks. For AAV8 transduction, AAV-TBG-Vector or AAV-TBG-*Gdf10* (1×10^11^ genome copies/mouse) was delivered by tail vein injection. For *Gdf10* knockdown in mice, mice were injected 5 x 10^7^ TU/mouse lentivirus particles via the tail vein. The sequence of the antisense shRNA of *Gdf10* was 5′- GCTACAGAGATACGACCCATT-3′. Recombinant human GDF10 (1 mg/kg), blank-LNP or *mGdf10*-LNP (5ug mRNA/mouse) were delivered four times by tail vein injection two weeks prior to sacrifice. The number of mice in each group is specified in the figure legend.

### Cell culture and treatment

HSCs were isolated from mice livers by collagenase digestion followed by gradient centrifugation, as previously described 23. Primary cultured HSCs were infected with a SV40 large T antigen-expressing retrovirus and were selected using G418 solution to obtain immortalized HSCs. Immortalized HSCs and LX2 cells were cultured in DMEM supplemented with 10% fetal bovine serum (FBS) and 100 μg/mL penicillin-streptomycin at 37 °C in an incubator with 5% CO2. Cells were treated with GDF10 recombinant protein (200 ng/ml; R&D Systems, 1543-BP-025) and TGFβ1 (10ng/ml novoprotein, CA59) for 24 or 48 hours. For *Gdf10* knockdown, 4 × 10^6^ TU lentivirus particles were used to transfect HSCs. To evaluate the effect of LNPs, activated HSCs after TGFβ1 treatment in a six-well plate (5 × 10^5^ per well) were incubated with *mGdf10* LNPs (50 nM) for 48 hours. For better preserving quiescence of HSCs, immortalized HSCs were cultured in DMEM supplemented with 2% FBS and 100 μg/mL penicillin-streptomycin at 37 °C in an incubator with 5% CO2.

### Plasma assays

The plasma alanine aminotransferase (ALT) and aspartate aminotransferase (AST) levels were measured using commercial kits from Nanjing Jianchen Bioengineering Institute (NJJCBIO, C009-2-1&C010-2-1) following the manufacturer's instructions. Plasma concentrations of cholesterol (T-CHO) and triglyceride (TG) were measured using assay kits from Nanjing Jianchen Bioengineering Institute (NJJCBIO, A111-1-1& A110-1-1).

### Glucose and insulin tolerance testing

For the glucose tolerance test (GTT), mice were fasted for 16 h and received 1 g/kg body weight D-glucose (Sigma-Aldrich, 1034122, dissolved in saline) by i.p. Blood samples were collected, and glucose levels were monitored with a portable glucometer at 0-, 15-, 30-, 60-, 90-, and 120-min time points after i.p. For the insulin tolerance test (ITT), mice were fasted for 5 h and received 0.5 U/kg body weight insulin (MedChem Express, HY-P0035, dissolved in saline) by i.p. Blood glucose levels were also measured at 0-, 15-, 30-, 60-, 90-, and 120-min time points after i.p.

### Histology and immunofluorescence staining

Liver tissues were fixed in 4% paraformaldehyde at 4 °C overnight, processed for paraffin embedding, and stained with hematoxylin and eosin (H&E). Sirius Red and Masson staining were performed as previously described to evaluate liver fibrosis 24. Liver tissues were fixed with 4% paraformaldehyde for 4 hours and subsequently embedded in OCT. Oil Red O was used to analyze intracellular triglyceride accumulation. Frozen sections were permeabilized with 0.3% Triton X-100 in PBS and then blocked in 5% bovine serum albumin (BSA), followed by incubation in primary antibody solution overnight at 4 °C, and subsequently in secondary antibody solution at room temperature for 2 hours. Sections were stained with DAPI (Invitrogen, 10236276001) for 10 minutes after washing with PBS, mounted with ProLong™ Gold Antifade Mountant (Thermo Fisher Scientific, P10144), and imaged with a fluorescence microscope. The following antibodies were used: anti-α-SMA (Proteintech, 14395-1-AP), anti-GDF10 (Bioss, bs-5720R), CoraLite488-conjugated Goat Anti Rabbit IgG (H+L) (Proteintech, SA00013-2), CoraLite594-conjugated Donkey Anti-Rabbit IgG (H+L) (Proteintech, SA00013-8).

### Gene expression analyses

Total RNA was extracted from snap-frozen mouse livers and cultured cells using TRIzol reagent (Vazyme Biotech, R401-01). 1 µg of total RNA was reverse-transcribed using MMLV-RT (Vazyme Biotech, R222-01), and qPCR was performed using SYBR Green Master Mix (Vazyme Biotech, Q311-02). Relative mRNA abundance was normalized to internal control genes encoding ribosomal protein 36B4. The sequences of the qPCR primers are listed in Supplementary Table S1.

RNA sequencing was performed using Illumina NovaSeq 6000 at GENEWIZ, INC SUZHOU. Raw sequencing read counts were normalized and analyzed using DESeq2 25. The differentially expressed genes were determined by FDRs < 0.05. RNA-seq data generated in this work have been deposited into the Gene Expression Omnibus (GEO) database (GSE303711).

### Immunoblotting analysis

Liver samples and cultured cells were harvested in RIPA buffer containing protease and phosphatase inhibitors freshly. All protein lysates were boiled for 10 minutes, separated by SDS-PAGE, and then transferred to PVDF membrane. For immunoblots, antibodies against α-SMA(Santa Cruz BioTech, sc-53142), GDF10 (ThermoFisher, PA5-70041), HSP90 (Proteintech, 13171-1-AP), GAPDH (Proteintech, 60004-1-Ig), COL1A1 (Proteintech, 14695-1-AP), SMAD2/3 (Santa Cruz BioTech, sc133098), Phospho SMAD2(Ser465/467) (Cell Signaling Technology, 3108T), Phospho SMAD3 (Ser423/425) (Cell Signaling Technology, 9520T), Phospho-p44/42 MAPK (Erk1/2) (Thr202/Tyr204) (Cell Signaling Technology, 4370T), p44/42 MAPK (Erk1/2) (Cell Signaling Technology, 4695T), TGFβ1 (abcam, ab179695), TβR1 (Santa Cruz BioTech, sc-518086), TβR2 (Santa Cruz BioTech, sc-17792), Peroxidase-AffiniPure Goat Anti-Rabbit IgG (H+L) (Jackson, 111-035-003), and Peroxidase AffiniPure Goat Anti-Mouse IgG (H+L) (Jackson, 115-035-003) were used.

### Cell co-culture experiments

To isolate hepatocytes, livers from AAV8-TBG-Vector, AAV8-TBG-GFP and AAV8-TBG-*Gdf10* treated mice were perfused with collagenase type IV (100 CDU/mL; Sigma, USA), and the liver was excised rapidly and placed into cold HBSS. The primary hepatocytes were released and filtered by a sterile 70-μm filter (Beyotime; FSTR070), and then centrifuged at 50 g for 5 min at 4 °C. The pellet was then resuspended in 20 mL of DMEM supplemented with 10% FBS for subsequent culture. The experiments were initiated with cells plated at the same density.

To determine secretion of GDF10 from hepatocytes and the crosstalk between hepatocytes and HSCs *in vitro*, primary hepatocytes were co-cultured with aHSCs using cell culture inserts (0.4 μm pore size, BIOFIL TCS016012). In brief, primary hepatocytes with *Gdf10* overexpression from AAV8-TBG-*Gdf10* treated mice were seeded in the upper chamber, while aHSCs were grown in the bottom chamber. The aHSCs and conditional medium of hepatocytes were collected for protein analysis after 48 h of co-culture, respectively.

### Nuclear translocation of SMAD2 analysis

Western blot and immunofluorescence staining were utilized to assess nuclear translocation of SMAD2. Before harvesting, cells were pretreated with GDF10 for 50 min, followed by stimulation with TGFβ1 for 50 min. Cell nuclei were isolated using Nuclear and Cytoplasmic Protein Extraction Kit (Sangon Biotech, C510001). In brief, cell pellets were resuspended in hypotonic lysis buffer. After centrifugation at 14,000 g for 5 minutes, the solution can be observed to separate into three distinct layers: a transparent lower layer, a white nuclear pellet in the middle layer, and a supernatant upper layer. Then, supernatant containing cytoplasmic proteins was collected. The white nuclear pellet was resuspended with nuclear extraction buffer and nuclear proteins were collected by centrifugation at 14,000 g for 5 minutes.

### Immunoprecipitation (IP)

To determine whether GDF10 can disrupt the interaction between TGFβ1 and TβR2, cells were treated with TGFβ1 in the absence or presence of different amounts of GDF10 as indicated, and TGFβ1/TβR2 interaction was then assessed. HSCs were serum-starved for 24 h and were pretreated with GDF10 for 1 hour prior to incubation with TGFβ1 at 10 ng/ml, then were collected at 1 hour after TGFβ1 treatment. Whole-cell lysates were prepared for immunoprecipitation analyses. Briefly, cell lysates were prepared and incubated overnight at 4 °C with IgG (proteintech, B900620), anti-TβR2 antibody (Santa Cruz BioTech, sc-17792), and Protein A/G Magnetic Beads (MCE, HY-K0202). Western blot was subsequently performed as described above.

### Metabolic analyses

Cells were treatment with GDF10 or TGFβ1 for 24 hours. Mitochondrial respiration and glycolytic flux were measured in the Seahorse XFe96 Analyzer (Agilent) using the Agilent Seahorse XF Glycolytic Rate Assay Kit in the presence of 1 μM rotenone/antimycin A and 100 mM 2DG, or the Agilent Seahorse XF Mito Stress Test Kit in the presence of 1.5 μM oligomycin, 3 μM FCCP, and 0.5 μM rotenone/antimycin A. All results were normalized to protein content. Data of oxygen consumption rate (OCR) during basal metabolism of Cell Mito Stress Test and glycolytic rate (GlycoPER) during basal metabolism of Glycolytic Rate Assay were used in the graphs depicting the quiescent or energetic state of HSCs under different conditions.

The levels of extracellular lactate and glucose consumption were measured in the media from GDF10-treated or TGFβ1-treated HSCs using a Lactic Acid Assay Kit and Glucose Kit (NJJCBIO, A019-2-1 & A154-1-1), following the manufacturer's instructions. All values were normalized to cell number.

### LNP studies

To obtain *Gdf10* mRNA, we retrieved complete coding sequence including signal peptide region from mouse gene library (NM_145741.3). *Gdf10* and luciferase mRNA were produced via *in vitro* transcription. Formulation was performed as previously described 26. An organic phase was composed of solubilizing ionizable lipid AA-T3A-C12 (MCE, HY-148859), cholesterol (Avanti Polar Lipids, #700100), DSPC (Avanti Polar Lipids, #850365), and C14-PEG2000 (Avanti Polar Lipids, #880150) at a molar ratio of 50: 38.5:10:1.5. The aqueous phase was prepared in 10 mM citrate buffer (pH 3) with mRNA. Then a microfluidic device was used to mix these two phases at an ionizable lipid: RNA weight ratio of 10:1, with a flow rate of 1.8 mL/min and 0.6 mL/min (3:1). Finally, LNPs were dialyzed against 1 × PBS for 2 h, filtered by a 0.22 μm filter, and stored at 4 °C. For characterization of LNP, Zetasizer Nano ZS90 was utilized to measured diameter and polydispersity index. mRNA encapsulation efficiency was determined by agarose gel electrophoresis.

For *in vivo* imaging system, luciferase mRNA LNP was intravenously injected into CCl4-induced fibrosis mice for 12 hours, and D-luciferin (150 mg/kg; Beyotime) was injected before animal live imaging detection.

### Data analysis

Published RNA sequencing (RNAseq) datasets were downloaded from the Gene Expression Omnibus database: single-cell RNAseq analysis of liver cells from chow and NASH diet-fed mice: GSE166504 27; single-cell RNAseq analysis of liver NPCs from chow and NASH diet-fed mice: GSE129516 23; single-cell RNAseq analysis of liver NPCs from chow, and CCl4 induced fibrosis mice: CRA007803 28; human liver bulk RNAseq analysis of individuals with healthy and cirrhosis: GSE25097 29. The UMAP plots, feature plots, violin plots, bubble plots, heatmaps, and correlation analysis were generated by R.

### Statistical analysis

All statistical analyses were conducted using GraphPad Prism 9 (GraphPad Software). Between-group comparisons were performed using two-tailed unpaired Student's t-tests. Statistical significance was defined as p< 0.05. Data are presented as mean ± SEM derived from at least three independent experiments, with significance levels denoted as follows: *p<0.05, **p<0.01 and ***p<0.001.

## Results

### GDF10 identifies as a HSCs regulator link to MASH progression

**Figure 1:**
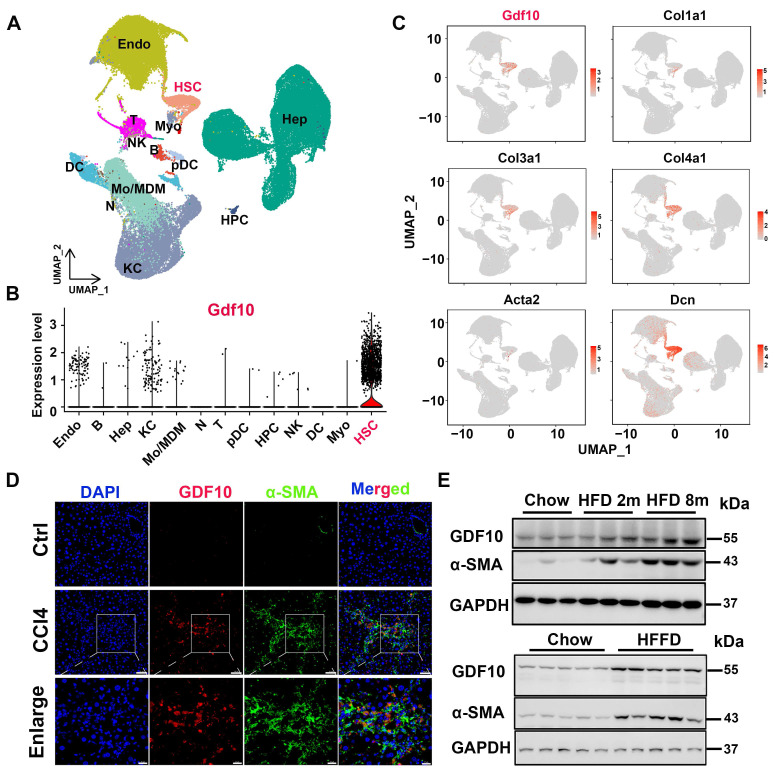
** GDF10 is special primarily expressed by HSCs in MASH.** A. UMAP visualization of liver cell clusters (GSE166504). B. Violin plots showing *Gdf10* gene expression for each cluster. C. UMAP visualization *Gdf10* and fibrosis-related gene mRNA levels in the liver. D. IF staining analysis of GDF10 and α-SMA protein levels in the liver, scale bars, 50 μm. E. Immunoblot analysis of GDF10 and α-SMA protein levels in the liver from chow, HFD- and HFFD-diet induced fibrotic liver (n = 3 for chow diet group, n = 3 for HFD 2m diet group, and n = 3 for HFD 8m diet group; n = 5 for chow diet group and n = 5 for HFFD diet group).

To broadly assess the regulators involved in liver fibrosis, we first analyzed single-cell datasets from mouse fibrotic livers induced by high fat/high fructose diet (HFHFD) (GSE166504) 27 and single-cell RNAseq analysis of liver NPCs from control and MASH mice (GSE129516). we found that *Gdf10* was predominantly expressed in hepatic stellate cells (HSCs) (Fig. 1A and B, S1A and B). Notably, *Gdf10* expression patterns closely aligned with those of established HSC markers in liver fibrosis (Fig. 1C and S1C). Immunofluorescence staining confirmed colocalization of GDF10 protein with α-SMA, a canonical HSC marker, in fibrotic liver tissues (Fig. 1D). Further characterization showed high GDF10 expression in non-parenchymal cells (NPCs), with minimal expression in hepatocytes (MPHs) - a pattern mirroring α-SMA distribution (Fig. S1D).

Importantly, *GDF10* expression was significantly elevated in human metabolic liver disease and fibrosis samples, showing strong correlation with classic fibrosis markers (*ACTA2*, *COL1A1*) (Fig. S1E). These findings were corroborated at the protein level across multiple experimental models, including diet-induced MASH and CCl4-induced fibrosis, where increased GDF10 expression coincided with enhanced extracellular matrix (ECM) deposition (Fig. 1E and S1F). Collectively, our results demonstrate that GDF10 is specifically expressed in HSCs and represents a potential biomarker for MASH progression.

### Overexpressing *Gdf10* ameliorates diet-induced MASH associated liver fibrosis

**Figure 2:**
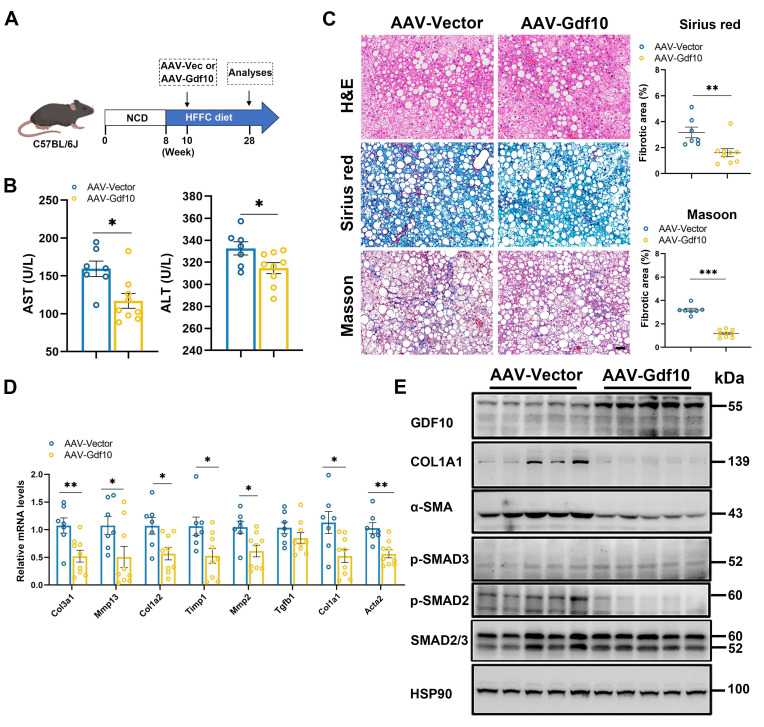
** Overexpressing *Gdf10* ameliorates diet-induced MASH associated liver fibrosis.** A. Experimental design for B-E. B. Measurement of serum AST and ALT levels of Ctrl and *Gdf10-OE* mice (n = 7 for control and n = 9 for *Gdf10-OE* group). C. Representative images of H&E, Sirius Red, and Masson staining, scale bars, 50 μm. D, E. qPCR (D) and immunoblot (E) analysis of indicated fibrosis-related genes in the liver of Ctrl and *Gdf10-OE* mice. Data are presented as mean ± SEM. *P < 0.05, **P < 0.01, ***P < 0.001.

To investigate whether *Gdf10* affects MASH progression, we administered control AAV or *Gdf10*-expressing AAV via tail vein injection to C57BL/6J mice in diet-induced MASH model (Fig. 2A). Hepatocyte-specific *Gdf10* overexpression via AAV-TBG-*Gdf10* elevates local GDF10 levels in the liver. AAV-mediated *Gdf10* delivery did not significantly alter body weight, liver-to-body weight ratio, or fat mass in HFFC diet-fed mice (Fig. S2A-C). Furthermore, *Gdf10* overexpression had no effect on glucose or lipid metabolic parameters (Fig. S2D-J). Notably, AAV-*Gdf10* treatment in HFFC diet-fed mice resulted in reduced serum AST and ALT levels, accompanied by significant improvements in hepatic fibrosis as demonstrated by H&E, Sirius Red, and Masson staining (Fig. 2B, C). Additionally, hepatic expression of fibrogenic genes was markedly decreased in *Gdf10*-overexpressing (*Gdf10*-*OE*) mice (Fig. 2D). Importantly, *Gdf10*-expressing AAV treatment inhibited the SMAD2-mediated liver fibrosis pathway in the HFFC diet-induced MASH model (Fig. 2E). These findings demonstrate that GDF10 attenuates liver fibrosis *in vivo* through mechanisms independent of glucose or lipid metabolism.

To further confirm the metabolic-independent anti-fibrotic effects of GDF10, we compared *Gdf10*-*OE* mice with wild-type (WT) littermates in both CCl4- and HFMCD diet-induced fibrosis models (Fig. S3A, S3K). Following three weeks of CCl4 administration, *Gdf10*-*OE* mice showed unchanged body weight (Fig. S3B), but reduced liver-to-body weight ratio compared to WT controls (Fig. S3C), with no alterations in blood glucose levels (Fig. S3D). Strikingly, AAV-*Gdf10* treatment in CCl4-induced fibrosis mice decreased serum AST and ALT levels and improved hepatic fibrosis, as evidenced by histological analyses (Fig. S3E-G). Both mRNA and protein levels of fibrogenic genes were reduced in the livers of *Gdf10*-*OE* mice (Fig. S3H-J).

In the HFMCD diet model, after 10 weeks of feeding, *Gdf10*-*OE* mice exhibited comparable body weight and liver-to-body weight ratios to WT mice (Fig. S3K-M) but showed reduced blood glucose and serum ALT levels (Fig. S3N and P). Histopathological assessment revealed significant improvement in liver fibrosis in *Gdf10*-*OE* mice (Fig. S3Q), which was further supported by decreased expression of fibrogenic genes at both mRNA and protein levels (Fig. S3R and S). Collectively, these data demonstrate that GDF10 effectively ameliorates diet-induced MASH associated liver fibrosis *in vivo*.

### *Gdf10* depletion accelerates liver fibrosis procession *in vivo*

**Figure 3:**
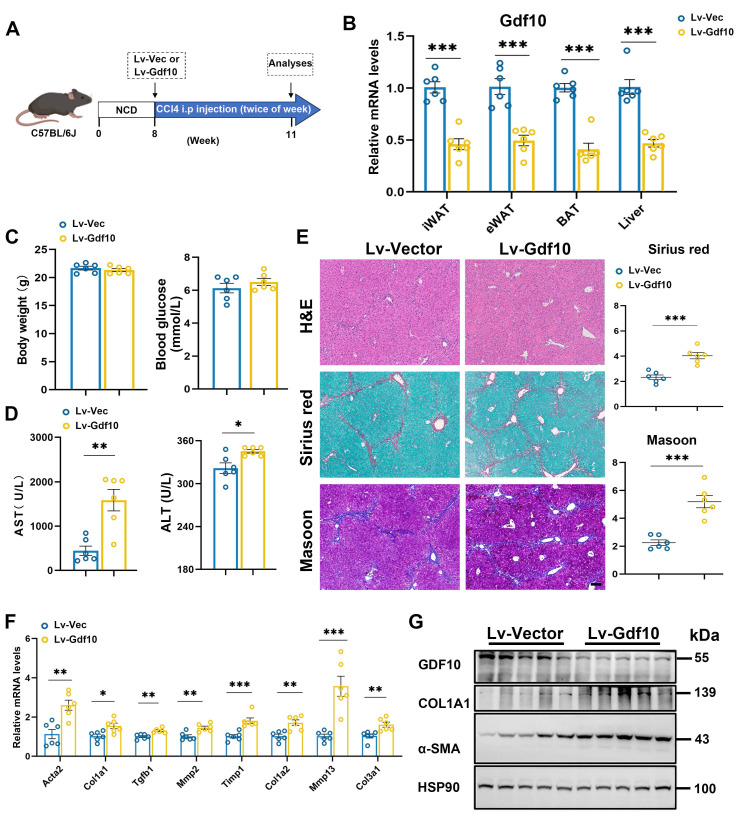
***Gdf10* depletion accelerates liver fibrosis procession *in vivo*.** A. Experimental design for B-G. B. qPCR analysis of *Gdf10* mRNA levels in multiple tissue from Ctrl (n = 6) and *Gdf10*-*KD* (n = 6) mice. C, D. Measurement of body weight, blood glucose (C), and serum AST and ALT (D) levels in Ctrl (n = 6 mice) and *Gdf10*-*KD* mice (n = 6 mice). E. Representative images of H&E, Sirius Red, and Masson staining in liver from Ctrl and *Gdf10*-*KD* mice, scale bars, 100 μm. F, G. qPCR (F) and immunoblot (G) analysis of indicated genes of the liver from Ctrl and *Gdf10*-*KD* mice. Data are presented as mean ± SEM. *P < 0.05, **P < 0.01, ***P < 0.001.

To further investigate the functional role of GDF10 in liver fibrosis progression, we employed a lentiviral system delivering shRNA to knock down *Gdf10* (*Gdf10*-*KD*) expression *in vivo*. Mice were given short-term injections of CCl4 (3 weeks) to induce liver fibrosis (Fig. 3A). The multiple tissue expression of GDF10 via Lv-sh*Gdf10* significantly decreased compared to the Lv-Vector control (Fig. 3B). Physiological assessments revealed no differences in body weight and blood glucose between groups (Fig. 3C), but notable increased in liver injury markers (AST, ALT) were observed (Fig. 3D). Histological analysis also showed that *Gdf10*-*KD* mice had exacerbated liver fibrosis compared to control mice (Fig. 3E). Consistently, mRNA and protein expression profiling showed upregulated levels of fibrotic genes in the Lv-sh*Gdf10* group, suggesting enhanced fibrotic activity, implicating stress-response involvement (Fig. 3F and G). These findings collectively highlight the role of GDF10 in regulating fibrotic procession in the liver.

### GDF10 attenuates hepatic fibrosis by suppressing TGFβ1-induced hepatic stellate cell activation

**Figure 4:**
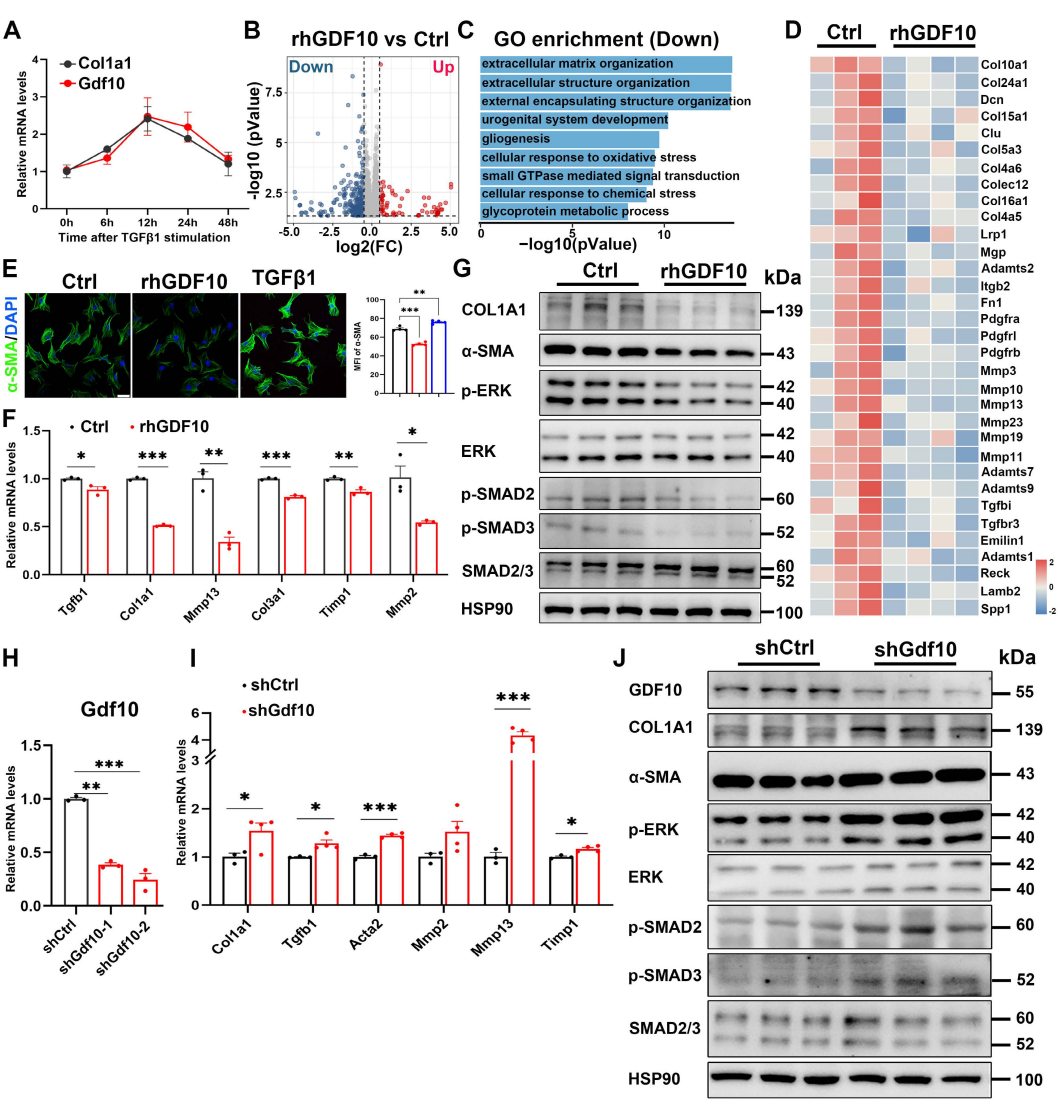
** GDF10 attenuates hepatic fibrosis by suppressing hepatic stellate cell activation.** A. qPCR analysis of *Gdf10* and *Col1a1* mRNA levels in HSCs during the culture activation. B. Volcano plot of differnential expressed genes in immortalized HSCs treated with rhGDF10 and vehicle. C. GO pathways enrichment analysis for downregulated pathways in immortalized HSCs treated with rhGDF10 compared to the control group. D. Heat map of fibrosis-related gene expression downregulated in immortalized HSCs with rhGDF10 treatment. E. IF staining analysis α-SMA protein level in immortalized HSCs with rhGDF10 or TGFβ1 treatment, respectively. scale bars, 50 μm. F, G. qPCR (F) and immunoblot (G) analysis of indicated genes in immortalized mouse HSCs with rhGDF10 treatment. H. qPCR analysis of *Gdf10* mRNA expression in immortalized HSCs transfected with Lv-Ctrl or Lv-sh*Gdf10*. I, J. qPCR (I) and immunoblot (J) analysis of indicated genes in immortalized HSCs infected with Lv-Ctrl or Lv-sh*Gdf10* (sh*Gdf10-2* in 6H) for 24 h. Data are representative of three independent experiments. Data are presented as mean ± SEM. *P < 0.05, **P < 0.01, ***P < 0.001.

Next, we investigated the significance of GDF10 in HSC activation. TGFβ1 primarily facilitates the transdifferentiation of HSCs into myofibroblast-like cells 30. We treated immortalized mouse HSCs with TGFβ1 protein. TGFβ1 protein treatment promotes time-dependent expression of *Col1a1* as well as *Gdf10* (Fig. 4A).

To gain insights into the molecular responses triggered by GDF10 in HSC activation, we performed RNA-sequencing (RNA-seq) analysis of immortalized mouse HSCs exposed to recombinant human GDF10 (rhGDF10). Among the 17,375 genes identified, 1,917 were differentially expressed in HSCs after rhGDF10 (Fig. 4B). Consistent with the anti-fibrotic role of GDF10, GO enrichment of genes down-regulated by rhGDF10 in immortalized mouse HSCs was dominated by extracellular-matrix-related processes (Fig. 4C), and the key pro-fibrotic collagens and ECM regulators were markedly blunted by exogenous rhGDF10 (Fig. 4D). Subsequently, immunoblot and immunofluorescence confirmed that rhGDF10 reduced fibrotic gene expression and phospho-SMAD2 and SMAD3 levels, whereas genetic silencing of *Gdf10* with shRNA (sh*Gdf10*) had the opposite effect, inhibiting activation of HSCs (Fig. 4E-J). Collectively, these data demonstrate that GDF10 suppresses the transcriptional and translational program driving ECM deposition and myofibroblast transition.

### Increased GDF10 levels shifts activated HSCs back towards a quiescent state

**Figure 5:**
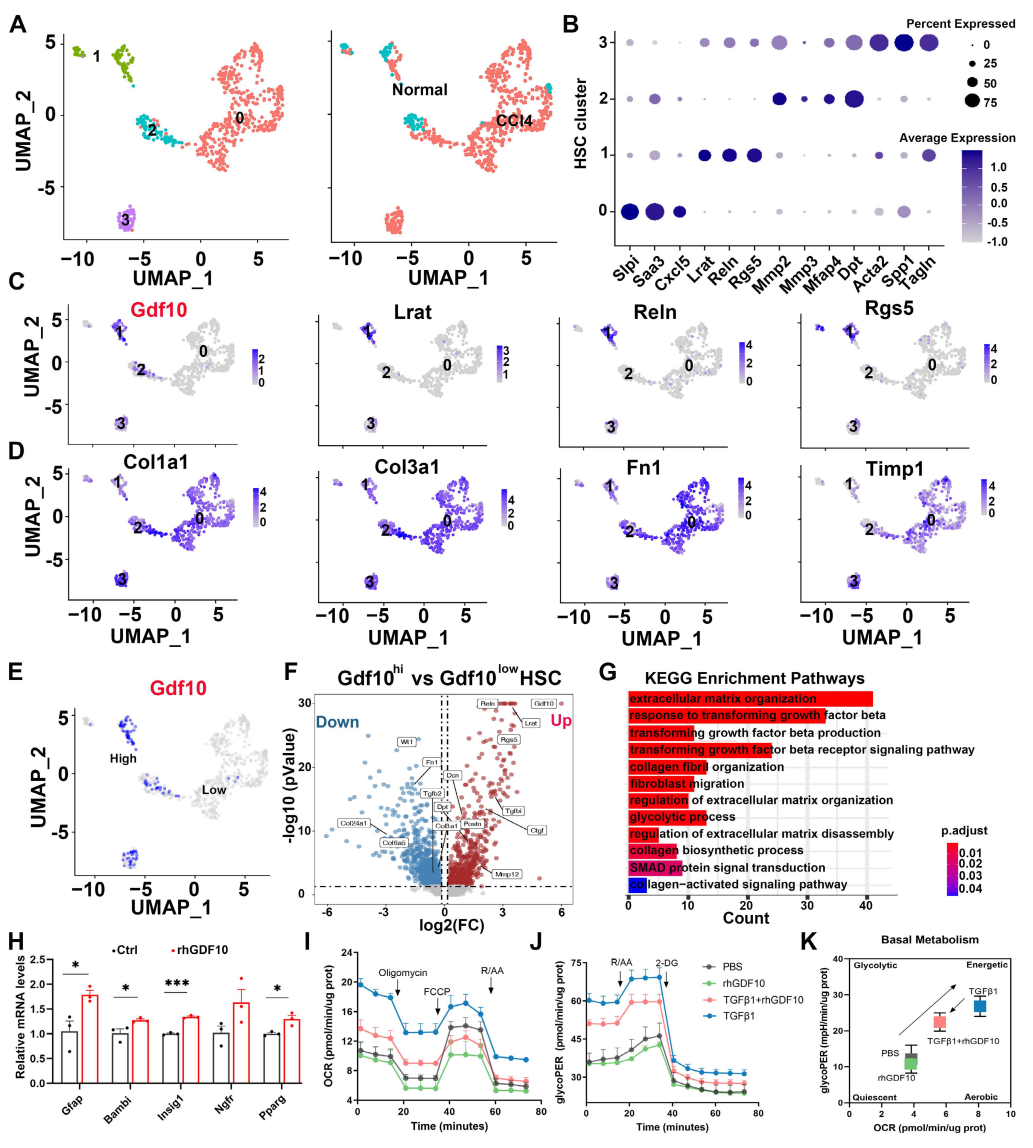
** Increased GDF10 levels shifts activated HSCs back towards a quiescent state.** A. UMAP showing the clustering of all HSCs subcluster from normal and CCl4 treated mouse livers (CRA007803). B. Dot plot of the expression of representative HSC marker genes. C. UMAP showing the representative *Gdf10* and HSCs marker genes of all four subclusters. D. UMAP showing the representative fibrosis-related genes of all four subclusters. E. UMAP visualization of the cellular expression of *Gdf10* genes in HSC cluster. F. Volcano plot of differential genes in *Gdf10^hi^* verse *Gdf10^low^* HSCs. G. Representative KEGG enriched with DEGs in HSCs from *Gdf10^hi^* verse *Gdf10^low^* HSCs. H. qPCR analysis of indicated genes in immortalized HSCs with rhGDF10 treatment. I, J. OCR (I) and GlycoPER (J) in immortalized HSCs with rhGDF10 or TGFβ1 treatment in OCR and GlycoPER process, respectively. K. Energy map showing the OCR vs GlycoPER ratio in immortalized HSCs with rhGDF10 or TGFβ1 treatment. Data are representative of three independent experiments and are presented as mean ± SEM. *P < 0.05, ***P < 0.001.

To further delineate the role of GDF10 in inhibiting liver fibrosis, we first analyzed scRNA-seq data of CCl4-induced mouse liver and it revealed that *Gdf10* was more specifically expressed in quiescent HSCs, with less coincidence with canonical aHSC markers (Fig. 5A-D). Furthermore, differential expression between *Gdf10^high^* and *Gdf10^low^* HSC subpopulations showed that *Gdf10^high^* cells exhibited enriched expression of quiescence-related genes and down-regulation of activation signatures (Fig. 5E-G). KEGG pathway analysis of these subclusters further linked *Gdf10* abundance to suppression of TGF-β receptor signaling, ECM organization, and collagen fibril formation (Fig. 5G; Fig. S4A). Also, rhGDF10 restored expression of qHSCs markers in immortalized HSCs (Fig. 5H; Fig. S4B-D). These results indicate that high endogenous GDF10 promotes toward a quiescent, anti-fibrotic HSC state.

Quiescent HSCs undergo activation and transdifferentiation into proliferative, motile myofibroblasts that secrete extracellular matrix-a process requiring rapid metabolic adaptation to meet increased energy demands 21, 31. This metabolic reprogramming predominantly relies on aerobic glycolysis 22, 32-35. To investigate GDF10's role in promoting HSC reversion to quiescence and associated metabolic remodeling, we conducted mechanistic studies using cultured cell models. Gene set enrichment analysis (GSEA) revealed significant alterations in the glycolysis pathway, with glycolysis-related genes being downregulated in GDF10-treated HSCs (Fig. S4E and F). Metabolic flux analyses demonstrated that GDF10 attenuated TGF-β-driven increases in basal glycolysis and compensatory glycolytic capacity, as measured by OCR and the glycolytic proton efflux rate (GlycoPER) (Fig. 5I-K; Fig. S4G-M). Consistently, GDF10 downregulated key glycolytic enzymes and blocked the metabolic reprogramming associated with HSC activation, revealing its dual function in restraining both ECM deposition and the glycolytic switch that drives myofibroblast transdifferentiation.

### GDF10 exerts liver fibrosis by competitively inhibiting TGF-β-SMAD 2/3 signaling

**Figure 6:**
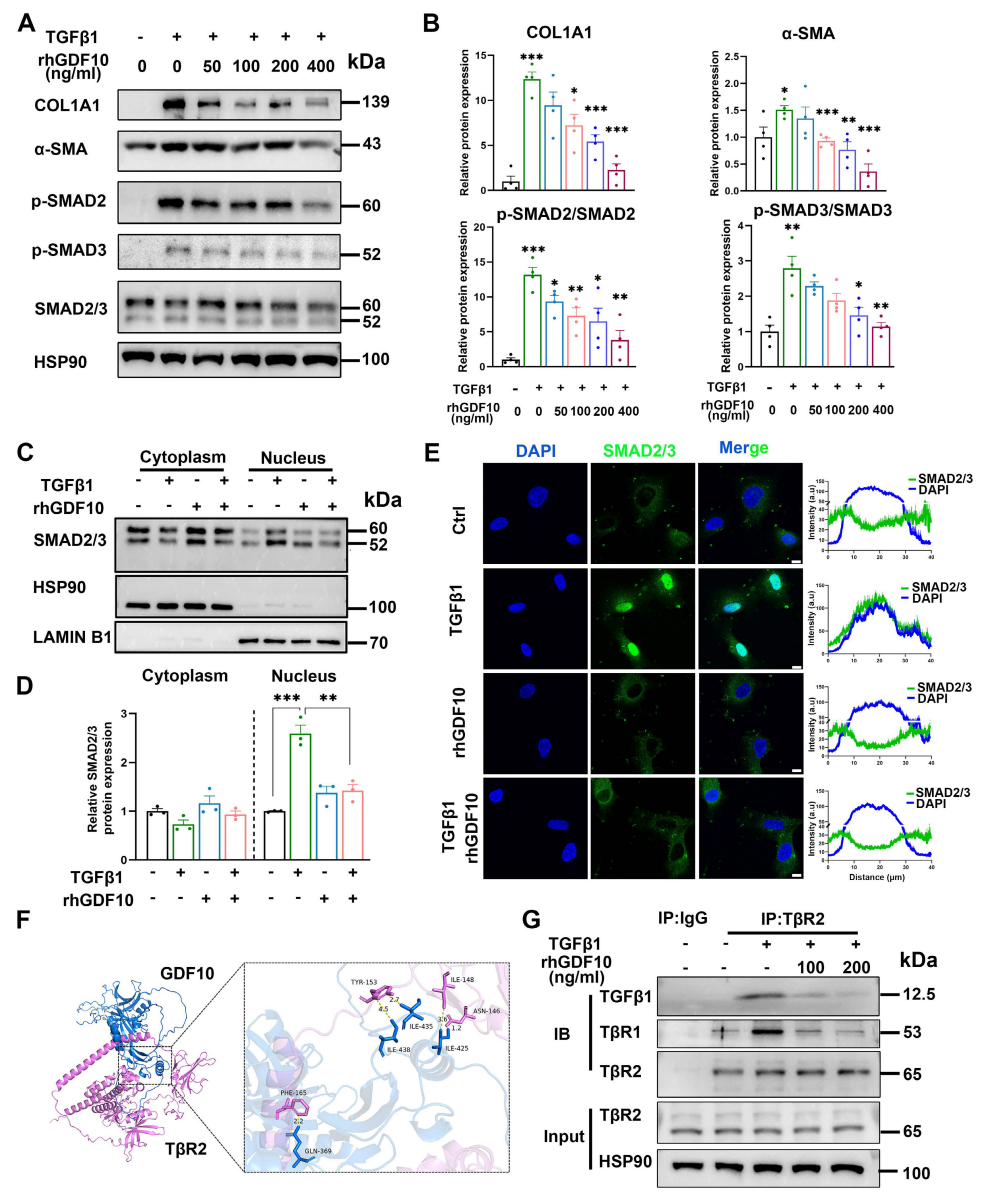
** GDF10 exerts anti-fibrotic effects by competitively inhibiting TGF-β-SMAD 2/3 signaling.** A, B. Immunoblot showing the levels of the indicated proteins in activated HSCs with difference dose of rhGDF10 treatment (A) and their qualification of relative protein expression (B). C-E. Immunoblot (C, D) and immunofluorescence staining (E) showing the levels of SMAD2/3 in cytoplasm and nucleus with TGFβ1 or rhGDF10 treatment. scale bars, 10 μm. F. Molecular docking prediction between the structure of mature GDF10 and the extracellular domains (ECD) of mouse TGFBR2 (GDF10: blue, TGFBR2: pink). G. GDF10 inhibited the interaction between TβR2 and TGFβ1 dose-dependently. HSCs were pre-incubated with different amounts of GDF10 for 1 h and then treated with TGFβ1 (10 ng/ml) for 1 h. Cells were collected and immunoprecipitated with anti-TβR2 or anti-IgG. Data are representative of three independent experiments and are presented as mean ± SEM. *P < 0.05, **P < 0.01, ***P < 0.001.

To define the antifibrotic mechanism of GDF10 at the cellular level, we interrogated its impact on immortalized HSCs. In line with its anti-fibrotic function, rhGDF10 dose-dependently blunted TGFβ1-induced COL1A1 and α-SMA expression (Fig. 6A and B). Furthermore, rhGDF10 blocked TGFβ1-triggered phosphorylation and nuclear translocation of SMAD2/3 (Fig. 6C-E), indicating direct interference with canonical TGF-β signaling. During HSC activation, TβR2 is the receptor for TGFβ1, recruiting TβR1 and leading to phosphorylation of SMAD2/3. Given that TGFβ1 and GDF10 belong to the same superfamily, whether they can compete for the identical receptor warrants further investigation. Molecular docking was used to predict if GDF10 binds to TβR2 (Fig. 6F). Mechanistically, co-immunoprecipitation revealed that GDF10 associates with TβR2 (Fig. 6G), suggesting competition with TGFβ1 for receptor engagement. Moreover, we found conditioned medium containing rhGDF10, but not control medium, significantly suppressed TGFβ1-induced COL1A1 and α-SMA up-regulation (Fig. S5B and C). In LX2 cells, rhGDF10 reduced p-SMAD2 levels, reinforcing its inhibitory effect on SMAD2/3 activation (Fig. S5D). To better preserve quiescence of HSCs, we also cultured HSCs with 2% FBS and obtained consistent results (Fig. S6). Essentially, co-culture experiments showed that hepatocyte-specific *Gdf10* overexpression via AAV-TBG-*Gdf10* elevated local GDF10 levels (Fig. S7A-C). Collectively, these data demonstrate that GDF10 antagonizes TGFβ1 signaling by targeting TβR2, thereby preventing SMAD2/3-mediated HSC activation and ECM production.

### LNP-encapsulated *mGdf10* retains bioactivity and further amplifies anti-fibrotic efficacy

**Figure 7:**
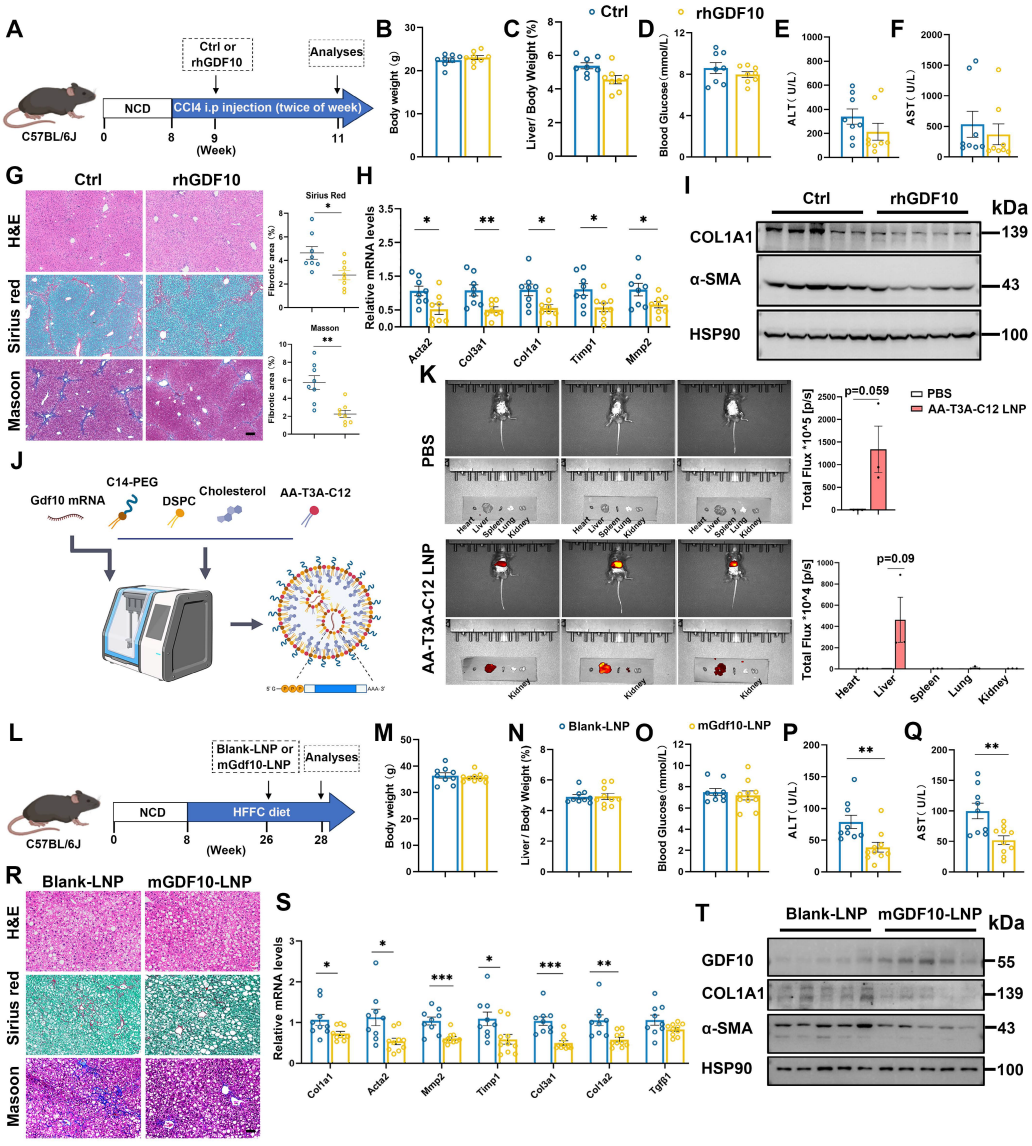
** rhGDF10 and LNP-encapsulated *mGdf10* exhibit anti-fibrotic effects.** A. Experimental design for B-I. Measurement of body weight (B), liver/body weight (C), blood glucose (D), serum ALT (E) and AST (F) levels in the CCl4-induced fibrosis mice treated with rhGDF10 treatment (n = 8 for control and n = 8 for rhGDF10 group). G. Representative images and qualification of H&E, Sirius Red, and Masson staining in liver from rhGDF10 treated mice, scale bars, 100 μm. H, I. qPCR (H) and immunoblot (I) analysis of indicated genes in liver from rhGDF10 treated mice. J. Schematic diagram of *mGdf10*-LNP production. K. *In vivo* imaging analysis of mice injected with *mGdf10*-LNP (n = 3 for control and n = 3 for *mGdf10*-LNP group). L. Experimental design for M-T. M-Q. Measurement of body weight (M), liver/body weight (N), blood glucose (O), serum ALT (P) and AST (Q) levels in the HFFC-diet fibrosis mice treated with *mGdf10*-LNP (n = 9 for control and n = 10 for *mGdf10*-LNP group). R. Representative images and qualification of H&E, Sirius Red, and Masson staining in liver from *mGdf10*-LNP treated mice, scale bars, 50 μm. S, T. qPCR (S) and immunoblot (T) analysis of indicated genes in liver from *mGdf10*-LNP treated mice. Data are presented as mean ± SEM. *P < 0.05, **P < 0.01, ***P < 0.001.

We identify GDF10 as a precise therapeutic agent capable of reversing fibrosis. Compared with vehicle-treated controls, systemic administration of rhGDF10 did not significantly alter body weight and liver-to-body weight ratio in CCl4-induced mice (Fig. 7A-C). Furthermore, rhGDF10 treatment did not affect blood glucose levels, and only slightly decreased serum ALT, or AST levels (Fig. 7D-F). However, it significantly reduced Sirius Red-positive fibrotic areas and downregulated the expression of fibrosis-related genes in CCl4-induced mice (Fig. 7G and H). Immunoblot of whole-liver lysates revealed significant down-regulation of COL1A1 and α-SMA (Fig. 7I), confirming potent inhibition of HSCs. These data establish that GDF10 attenuates liver fibrosis through pathways that are mechanistically separable from its effects on glucose or lipid homeostasis.

To translate this anti-fibrotic activity into a clinically viable modality, we encapsulated mouse *Gdf10* mRNA in an ionizable lipid nanoparticle (LNP) composed of DSPC, cholesterol and the novel ionizable lipid AA-T3A-C12 (Fig. 7J). As described by Han *et al.*^26^, AA-T3-C12 LNP was developed to target sigma receptors of fibroblasts and aHSCs, exhibiting high specificity. Physicochemical characterization showed a mean diameter of 100 nm, with a high RNA encapsulation efficiency (Fig. S8A and S8B). *In vitro* treatment of activated HSCs with *mGdf10*-LNP yielded robust GDF10 protein expression and suppressed COL1A1 and α-SMA (Fig. S8C). We then administered LNP to mice and evaluated its tissue distribution. *In vivo* imaging revealed that following the injection of AA-T3-C12 LNPs, the particles predominantly accumulated in the liver, with faint detectable signals observed in other tissues (Fig. 7K). Consistently, qPCR and immunoblot analyses showed marked changes specifically in liver tissue (Fig. S8D and E). Furthermore, immunofluorescence analysis indicated pronounced alterations specifically in HSCs (Fig. S8F). Collectively, these data support the HSCs-targeting specificity of *mGdf10*-LNP. To evaluate the therapeutic efficacy, we administered *mGdf10*-LNP to mice subjected to either HFFC-diet-induced fibrosis or CCl4-induced liver injury (Fig. 7L and S8G). *mGdf10*-LNP treatment did not alter body weight, liver-to-body weight ratio or fasting glucose levels (Fig. 7M-O; Fig. S8H-J). By contrast, serum AST and ALT concentrations were markedly reduced after *mGdf10*-LNP administration (Fig. 7P and Q; Fig. S8K-L). Moreover, histological staining and fibrosis-related gene expression showed concordant reductions following *mGdf10*-LNP administration (Fig. 7R-T and S8M-O). Together, these data established LNP-mediated delivery system for *Gdf10*, translating the cytokine's anti-fibrotic activity into a practical therapeutic modality.

## Discussion

The discovery of GDF10 as a HSC-derived factor capable of attenuating liver fibrosis through dual modulation of TGF-β signaling and metabolic reprogramming represents a significant advancement in our understanding of antifibrotic mechanisms. Our findings position GDF10 as a master regulator of HSC quiescence, with its expression inversely correlated with fibrotic activation across multiple animal models and human cirrhotic livers. This aligns with emerging evidence highlighting the remarkable plasticity of HSCs and their capacity to revert to a quiescent state during fibrosis regression 16, 36. The specificity of GDF10 action on HSCs, achieved through competitive inhibition of TGFβ1 binding to TβR2, offers distinct advantages over broad-spectrum antifibrotic approaches. This mechanism resembles the decoy receptor strategy but operates with endogenous precision, avoiding the off-target effects commonly associated with global TGF-β pathway inhibition 14, 37. The downstream consequences of this interaction-suppression of SMAD2/3 phosphorylation and nuclear translocation-effectively disrupt the canonical fibrogenic cascade while preserving the indispensability of TGF-β signaling in other cell types.

The metabolic effects of GDF10 on HSCs add another layer of complexity to its antifibrotic action. Our data demonstrate that GDF10 reverses the glycolytic switch characteristic of activated HSCs, a metabolic adaptation increasingly recognized as critical for myofibroblast persistence 21, 22. This process of metabolic reprogramming involves downregulation of key glycolytic enzymes and restoration of oxidative phosphorylation, mirroring the metabolic profile of quiescent HSCs. The connection between metabolic flexibility and fibrogenic potential has gained considerable attention in recent years, with studies highlighting how metabolic intermediates can directly influence epigenetic modifications and transcriptional programs driving fibrosis 31, 38. Our findings that GDF10 simultaneously targets both the TGF-β signaling axis and metabolic reprogramming suggest it acts at a critical nodal point coordinating these two fundamental aspects of HSC activation. This dual mechanism may explain its superior efficacy compared to approaches targeting either pathway alone, as evidenced by the robust fibrosis regression observed across multiple animal models without metabolic perturbations.

The translational potential of our work is underscored by the development of liver-targeted LNPs for *Gdf10* mRNA delivery, which addresses a major challenge in antifibrotic therapy-achieving sufficient drug concentrations in HSCs while minimizing systemic exposure. The success of this delivery platform builds upon recent advances in nucleic acid therapeutics and nanomedicine 39, 40, demonstrating how rational nanoparticle design can overcome biological barriers to achieve cell-type specific delivery. Importantly, our LNP formulation-maintained HSCs specificity without detectable accumulation in other organs and other liver cells, a crucial feature for chronic therapy in fibrotic diseases. The absence of weight or glucose perturbations following *mGdf10*-LNP treatment contrasts sharply with existing pharmacologic approaches such as FXR agonists 41, 42. This favorable safety profile, combined with potent antifibrotic efficacy, positions *mGdf10*-LNP as a promising candidate for clinical translation.

Our study also raises important questions about the broader biological roles of GDF10 in liver physiology and disease. While we focused on its antifibrotic effects, GDF10's expression pattern and mechanism of action suggest potential involvement in other aspects of liver homeostasis and repair. The observation that *GDF10* expression correlates with fibrosis severity in human cirrhosis hints at its possible role as an endogenous brake on fibrogenesis, potentially explaining why some patients develop progressive fibrosis while others maintain stable disease 1, 7. Furthermore, the interplay between GDF10 and other resident liver cells, particularly Kupffer cells and hepatocytes, warrants further investigation given their known contributions to fibrosis progression through inflammatory signaling and oxidative stress 43, 44. Thus, whether GDF10 has immunomodulatory functions that complement its direct effects on HSCs, and the upstream mechanism of GDF10 require further investigation.

## Conclusions

Our work establishes GDF10 as a pivotal regulator of HSC biology that integrates TGF-β signaling inhibition with metabolic reprogramming to promote fibrosis resolution. The development of a targeted LNP-mRNA delivery system not only validates GDF10's therapeutic potential but also provides a blueprint for RNA-based treatment of fibrotic diseases. These findings advance our fundamental understanding of HSC plasticity while offering a clinically translatable strategy that addresses the urgent unmet need for effective antifibrotic therapies. As the field moves toward precision medicine approaches for liver disease, GDF10 emerges as both a promising therapeutic agent and a valuable tool for elucidating the complex interplay between signaling pathways and cellular metabolism in fibrosis pathogenesis.

## Supplementary Material

Supplementary figures and table.

## Abbreviations

GDF10: growth differentiation factor 10
AAV: adenovirus-associated virus
α-SMA: alpha-smooth muscle actin
CCl4: carbon tetrachloride
ECM: extracellular matrix
HSCs: hepatic stellate cells
MASLD: metabolic dysfunction-associated steatotic liver disease
MASH: metabolic dysfunction-associated steatohepatitis
NPCs: nonparenchymal cells
MPHs: mouse primary hepatocytes
TBG: thyroxine binding globulin
TGF: transforming growth factor
UMAP: uniform manifold approximation and projection
LNP: lipid nanoparticle

## References

[1] Huang DQ, Wong VWS, Rinella ME, Boursier J, Lazarus JV, Yki-Järvinen H (2025). Metabolic dysfunction-associated steatotic liver disease in adults. Nat Rev Dis Primers.

[2] Ning B, Wang SA, Young MJ, Chen YC, Hung Y, Huong TT (2025). USP24 upregulation stabilizes PKA-Cα to promote lipogenesis, inflammation, and fibrosis during MASH progression. J Biomed Sci.

[3] Wang S, Friedman SL (2023). Found in translation-Fibrosis in metabolic dysfunction-associated steatohepatitis (MASH). Sci Transl Med.

[4] Schwabe RF, Tabas I, Pajvani UB (2020). Mechanisms of Fibrosis Development in Nonalcoholic Steatohepatitis. Gastroenterology.

[5] Jeong BK, Choi WI, Choi W, Moon J, Lee WH, Choi C (2024). A male mouse model for metabolic dysfunction-associated steatotic liver disease and hepatocellular carcinoma. Nat Commun.

[6] Younossi ZM, Koenig AB, Abdelatif D, Fazel Y, Henry L, Wymer M (2016). Global epidemiology of nonalcoholic fatty liver disease-Meta-analytic assessment of prevalence, incidence, and outcomes. Hepatology.

[7] Angulo P, Kleiner DE, Dam-Larsen S, Adams LA, Bjornsson ES, Charatcharoenwitthaya P (2015). Liver Fibrosis, but No Other Histologic Features, Is Associated With Long-term Outcomes of Patients With Nonalcoholic Fatty Liver Disease. Gastroenterology.

[8] Do A, Zahrawi F, Mehal WZ (2025). Therapeutic landscape of metabolic dysfunction-associated steatohepatitis (MASH). Nat Rev Drug Discov.

[9] Tsuchida T, Friedman SL (2017). Mechanisms of hepatic stellate cell activation. Nat Rev Gastroenterol Hepatol.

[10] Zhang M, Wu Z, Salas SS, Aguilar MM, Trillos-Almanza MC, Buist-Homan M (2023). Arginase 1 expression is increased during hepatic stellate cell activation and facilitates collagen synthesis. J Cell Biochem.

[11] Abdulmajeed RJ, Sergi CM (2025). Liver Sinusoidal Endothelial Cells and Their Regulation of Immunology, Collagenization, and Bioreactivity in Fatty Liver: A Narrative Review. Int J Mol Sci.

[12] Kumar S, Duan Q, Wu R, Harris EN, Su Q (2021). Pathophysiological communication between hepatocytes and non-parenchymal cells in liver injury from NAFLD to liver fibrosis. Adv Drug Deliv Rev.

[13] Meng XM, Nikolic-Paterson DJ, Lan HY (2016). TGF-β: the master regulator of fibrosis. Nat Rev Nephrol.

[14] Derynck R, Zhang YE (2003). Smad-dependent and Smad-independent pathways in TGF-beta family signalling. Nature.

[15] Liu Z, Li C, Kang N, Malhi H, Shah VH, Maiers JL (2019). Transforming growth factor β (TGFβ) cross-talk with the unfolded protein response is critical for hepatic stellate cell activation. J Biol Chem.

[16] Kisseleva T, Brenner D (2021). Molecular and cellular mechanisms of liver fibrosis and its regression. Nat Rev Gastroenterol Hepatol.

[17] Schwabe RF, Brenner DA (2025). Hepatic stellate cells: balancing homeostasis, hepatoprotection and fibrogenesis in health and disease. Nat Rev Gastroenterol Hepatol.

[18] Li S, Nie EH, Yin Y, Benowitz LI, Tung S, Vinters HV (2015). GDF10 is a signal for axonal sprouting and functional recovery after stroke. Nat Neurosci.

[19] Upadhyay G, Yin Y, Yuan H, Li X, Derynck R, Glazer RI (2011). Stem cell antigen-1 enhances tumorigenicity by disruption of growth differentiation factor-10 (GDF10)-dependent TGF-beta signaling. Proc Natl Acad Sci U S A.

[20] Platko K, Lebeau PF, Byun JH, Poon SV, Day EA, MacDonald ME (2019). GDF10 blocks hepatic PPARγ activation to protect against diet-induced liver injury. Mol Metab.

[21] Trivedi P, Wang S, Friedman SL (2021). The Power of Plasticity-Metabolic Regulation of Hepatic Stellate Cells. Cell Metab.

[22] Mejias M, Gallego J, Naranjo-Suarez S, Ramirez M, Pell N, Manzano A (2020). CPEB4 Increases Expression of PFKFB3 to Induce Glycolysis and Activate Mouse and Human Hepatic Stellate Cells, Promoting Liver Fibrosis. Gastroenterology.

[23] Xiong X, Kuang H, Ansari S, Liu T, Gong J, Wang S (2019). Landscape of Intercellular Crosstalk in Healthy and NASH Liver Revealed by Single-Cell Secretome Gene Analysis. Mol Cell.

[24] Hong T, Xiong X, Chen Y, Wang Q, Fu X, Meng Q (2023). Parathyroid hormone receptor-1 signaling aggravates hepatic fibrosis through upregulating cAMP response element-binding protein-like 2. Hepatology.

[25] Love MI, Huber W, Anders S (2014). Moderated estimation of fold change and dispersion for RNA-seq data with DESeq2. Genome Biol.

[26] Han X, Gong N, Xue L, Billingsley MM, El-Mayta R, Shepherd SJ (2023). Ligand-tethered lipid nanoparticles for targeted RNA delivery to treat liver fibrosis. Nat Commun.

[27] Su Q, Kim SY, Adewale F, Zhou Y, Aldler C, Ni M (2021). Single-cell RNA transcriptome landscape of hepatocytes and non-parenchymal cells in healthy and NAFLD mouse liver. iScience.

[28] Cheng S, Zou Y, Zhang M, Bai S, Tao K, Wu J (2023). Single-cell RNA sequencing reveals the heterogeneity and intercellular communication of hepatic stellate cells and macrophages during liver fibrosis. MedComm (2020).

[29] Sung WK, Zheng H, Li S, Chen R, Liu X, Li Y (2012). Genome-wide survey of recurrent HBV integration in hepatocellular carcinoma. Nat Genet.

[30] Arumugam S, Li B, Boodapati SLT, Nathanson MH, Sun B, Ouyang X (2023). Mitochondrial DNA and the STING pathway are required for hepatic stellate cell activation. Hepatology.

[31] Horn P, Tacke F (2024). Metabolic reprogramming in liver fibrosis. Cell Metab.

[32] Fondevila MF, Novoa E, Gonzalez-Rellan MJ, Fernandez U, Heras V, Porteiro B (2024). p63 controls metabolic activation of hepatic stellate cells and fibrosis via an HER2-ACC1 pathway. Cell Rep Med.

[33] Wang F, Chen L, Kong D, Zhang X, Xia S, Liang B (2024). Canonical Wnt signaling promotes HSC glycolysis and liver fibrosis through an LDH-A/HIF-1α transcriptional complex. Hepatology.

[34] Bates J, Vijayakumar A, Ghoshal S, Marchand B, Yi S, Kornyeyev D (2020). Acetyl-CoA carboxylase inhibition disrupts metabolic reprogramming during hepatic stellate cell activation. J Hepatol.

[35] Gilgenkrantz H, Mallat A, Moreau R, Lotersztajn S (2021). Targeting cell-intrinsic metabolism for antifibrotic therapy. J Hepatol.

[36] Schwabe RF, Tacke F, Sugimoto A, Friedman SL (2025). Antifibrotic therapies for metabolic dysfunction-associated steatotic liver disease. JHEP Rep.

[37] Miyazawa K, Miyazono K (2017). Regulation of TGF-β Family Signaling by Inhibitory Smads. Cold Spring Harb Perspect Biol.

[38] Tian W, Hao C, Fan Z, Weng X, Qin H, Wu X (2015). Myocardin related transcription factor A programs epigenetic activation of hepatic stellate cells. J Hepatol.

[39] Akinc A, Maier MA, Manoharan M, Fitzgerald K, Jayaraman M, Barros S (2019). The Onpattro story and the clinical translation of nanomedicines containing nucleic acid-based drugs. Nat Nanotechnol.

[40] Chandler M, Johnson B, Khisamutdinov E, Dobrovolskaia MA, Sztuba-Solinska J, Salem AK (2021). The International Society of RNA Nanotechnology and Nanomedicine (ISRNN): The Present and Future of the Burgeoning Field. ACS Nano.

[41] Wang K, Zhang Y, Wang G, Hao H, Wang H (2024). FXR agonists for MASH therapy: Lessons and perspectives from obeticholic acid. Med Res Rev.

[42] Kremoser C (2021). FXR agonists for NASH: How are they different and what difference do they make?. J Hepatol.

[43] Krenkel O, Puengel T, Govaere O, Abdallah AT, Mossanen JC, Kohlhepp M (2018). Therapeutic inhibition of inflammatory monocyte recruitment reduces steatohepatitis and liver fibrosis. Hepatology.

[44] Gaul S, Leszczynska A, Alegre F, Kaufmann B, Johnson CD, Adams LA (2021). Hepatocyte pyroptosis and release of inflammasome particles induce stellate cell activation and liver fibrosis. J Hepatol.

