# Supplementary material for: GDF10 attenuates MASH progression by restoring quiescent hepatic stellate cells via competitive inhibition of TGF-β/SMAD2 signaling
Source: Int J Biol Sci. 2025 Oct 27;21(15):6997–7012. doi: 10.7150/ijbs.123784 (PMC12631229; doi:10.7150/ijbs.123784)
Supplement: Supplementary file 1 — Supplementary figures and table. [file ijbsv21p6997s1.pdf]

Supplementary material

GDF10 attenuates MASH progression by restoring quiescent hepatic stellate cells via competitive inhibition of TGF-β/SMAD2 signaling

Yajie Peng[1], Hongyan Lei[1], Jiahui Zhao[1], Huajuan Wang[1], Zheng Luo[1], Dixin Wang[1], Shujun Shi[1], Tianyi Wang[1], Jin Li[2], Zhiqing Pang[3], Bo Wang[1*], Xuelian Xiong[1,4*]

[1] Ministry of Education Key Laboratory of Metabolism and Molecular Medicine, Department of Endocrinology and Metabolism, Zhongshan Hospital, Fudan University, 200020, Shanghai, China

[2] State Key Laboratory of Genetic Engineering, School of Life Sciences, Fudan University, 200438, Shanghai, China

[3] Key Laboratory of Smart Drug Delivery, Ministry of Education, School of Pharmacy, Fudan University, 201203, Shanghai, China

[4] Department of Endocrinology, Xinhua Hospital Affiliated to Shanghai Jiao Tong University School of Medicine, 200092, Shanghai, China

*Corresponding authors: Xuelian Xiong; Bo Wang

E-mails: xuelian@fudan.edu.cn; wangboo7370@163.com

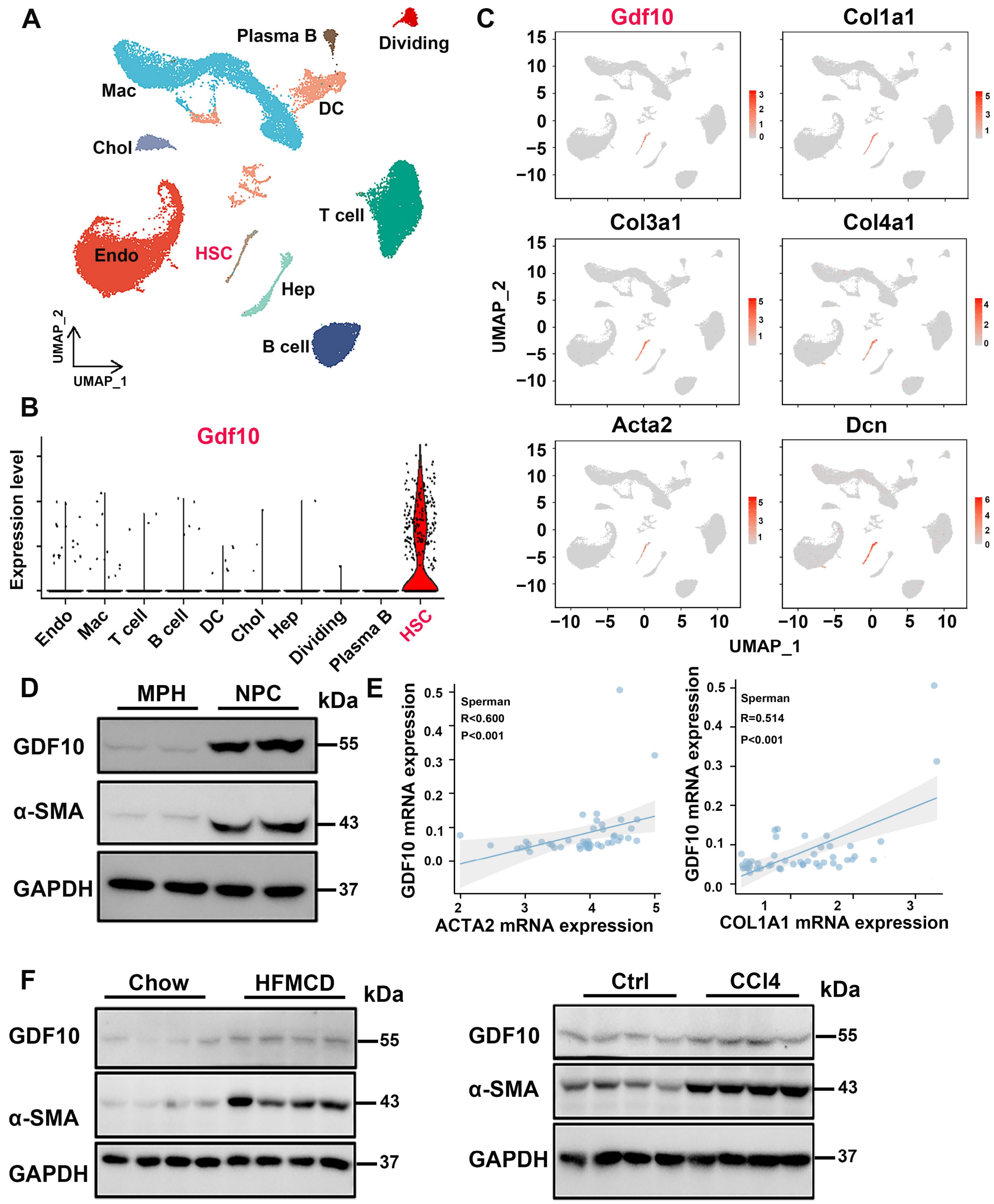

Figure S1. GDF10 is predominantly secreted by HSCs in MASH and correlates with disease progression, related to Figure 1. A. UMAP visualization of liver cell clusters (GSE129516). B. Violin plots showing *Gdf10* gene expression for each cluster. C. UMAP visualization *Gdf10* and fibrosis-related gene mRNA levels in the liver. D. Immunoblot analysis of GDF10 and α-SMA in MPH or NPCs, respectively. E. Linear regression for *GDF10* with *ACTA2* or *COL1A1* mRNA expression as a covariate in the human liver cirrhosis (GSE25097). F. Immunoblots analysis of GDF10 and α-SMA in

liver from HFMCD- or CCl4-induced fibrosis mice, respectively (n = 4 for chow diet group and n = 4 for HFMCD diet group; n = 4 for control and n = 4 for CCl4 group).

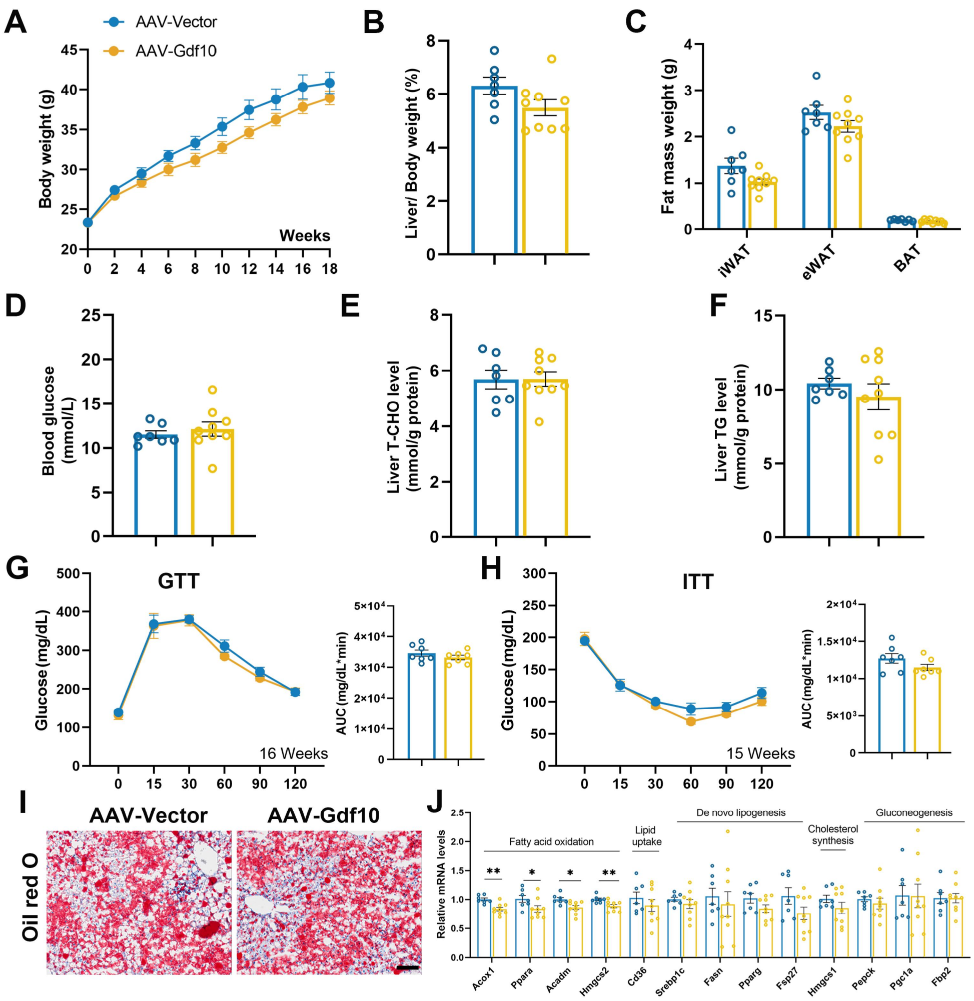

Figure S2. *Gdf10* overexpression had no effect on glucose or lipid metabolism parameters, related to figure 2. A-C. Measurement of body weight (A), liver/body weight, fat mass levels in HFFC-diet MASH mice treated with *Gdf10-OE* virus (n = 7 for control and n = 9 for *Gdf10-OE* group). D-F. Blood glucose levels (D), liver T-CHO levels (E), and the concentrations of liver TG (F) were evaluated in HFFC-diet MASH mice treated with *Gdf10-OE* virus (n = 7 for control and n = 9 for *Gdf10-OE* group). G, H. GTT and ITT were performed in mice after 16- or 15-weeks intervention, respectively (n = 6 for control and n = 7 for *Gdf10-OE* group). The area under the curve of GTT (G) and ITT (H) were measured. I. Representative images of oil red O staining of liver tissue in mice, scale bars, 100 μm. J. qPCR analysis of indicated genes of the liver in Ctrl and *Gdf10-OE* mice (n = 7 for control and n = 9 for *Gdf10-OE* group). Data are presented as mean ± SEM. *P < 0.05, **P < 0.01.

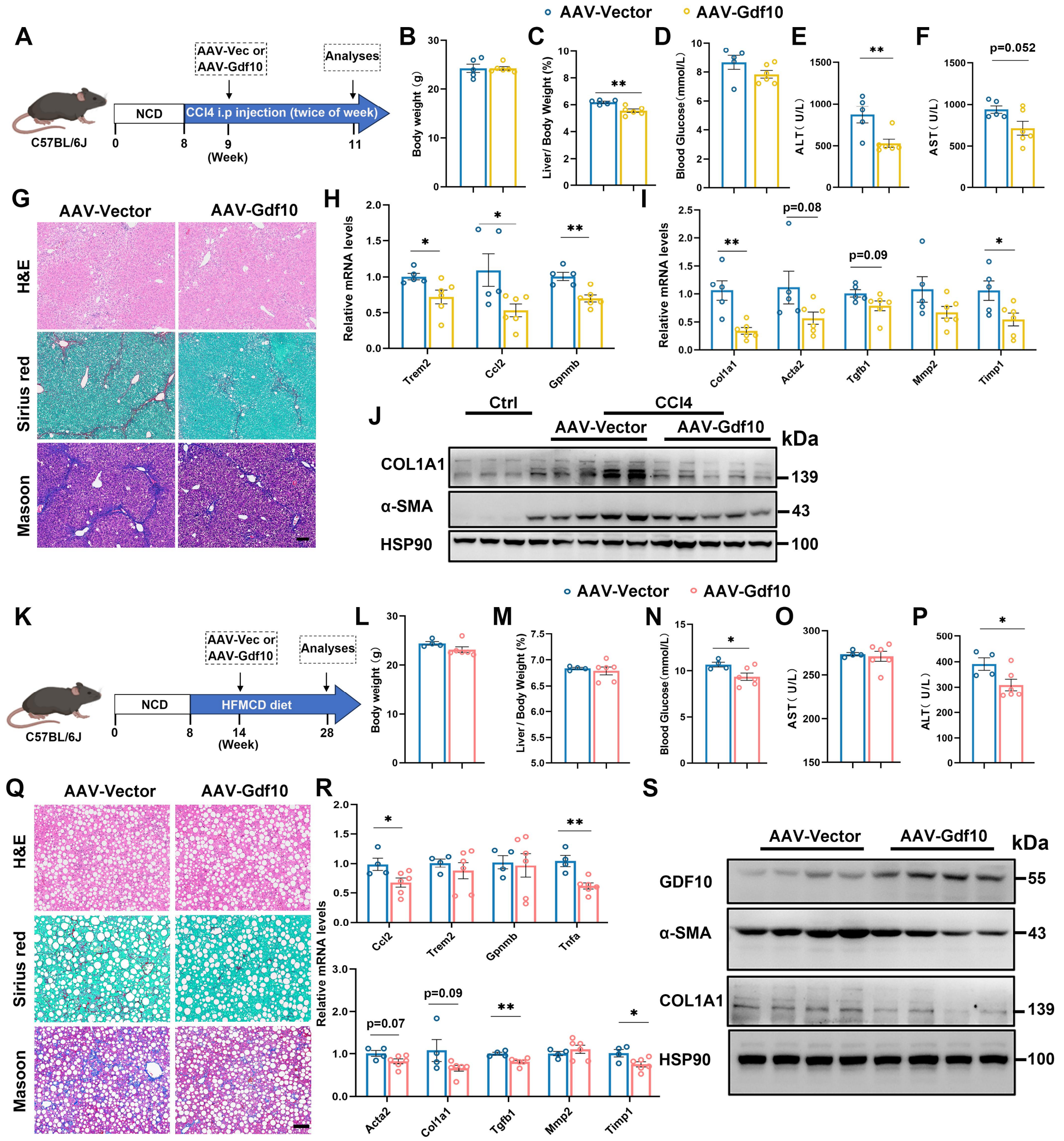

Figure S3. *Gdf10* overexpression attenuates liver fibrosis in CCl4-induced fibrosis mice and diet-induced MASH models, related to figure 2. A. Experimental design for B-J. B-F. Measurement of body weight (B), liver/body weight (C), blood glucose levels (D), serum ALT (E) and AST (F) in the CCl4-induced fibrosis mice treated with *Gdf10-OE* virus (n = 5 for control and n = 5 for *Gdf10-OE* group). G. Representative images and qualification of H&E, Sirius Red, and Masson staining in CCl4-induced fibrosis mice treated with *Gdf10-OE* virus. scale bars, 100 μm. H-J. qPCR and analysis of indicated genes in fibrosis-related genes mRNA (H, I) and protein (J) levels of the liver in CCl4-induced fibrosis mice treated with *Gdf10-OE* virus. K. Experimental design for L-S. L-P. Measurement of body weight (L), liver/body weight (M), blood

glucose levels (N), serum AST (O) and ALT (P) in the HFMCD-diet MASH mice treated with *Gdf10-OE* virus (n = 4 for control and n = 6 for *Gdf10-OE* group). Q. Representative images and qualification of H&E, Sirius Red, and Masson staining in the HFMCD-diet MASH mice treated with *Gdf10-OE* virus. scale bars, 100 µm. R, S. qPCR (R) and immunoblot (S) analysis of indicated genes of the liver in CCl4-induced fibrosis mice treated with *Gdf10-OE* virus. Data are presented as mean ± SEM. *P < 0.05, **P < 0.01.

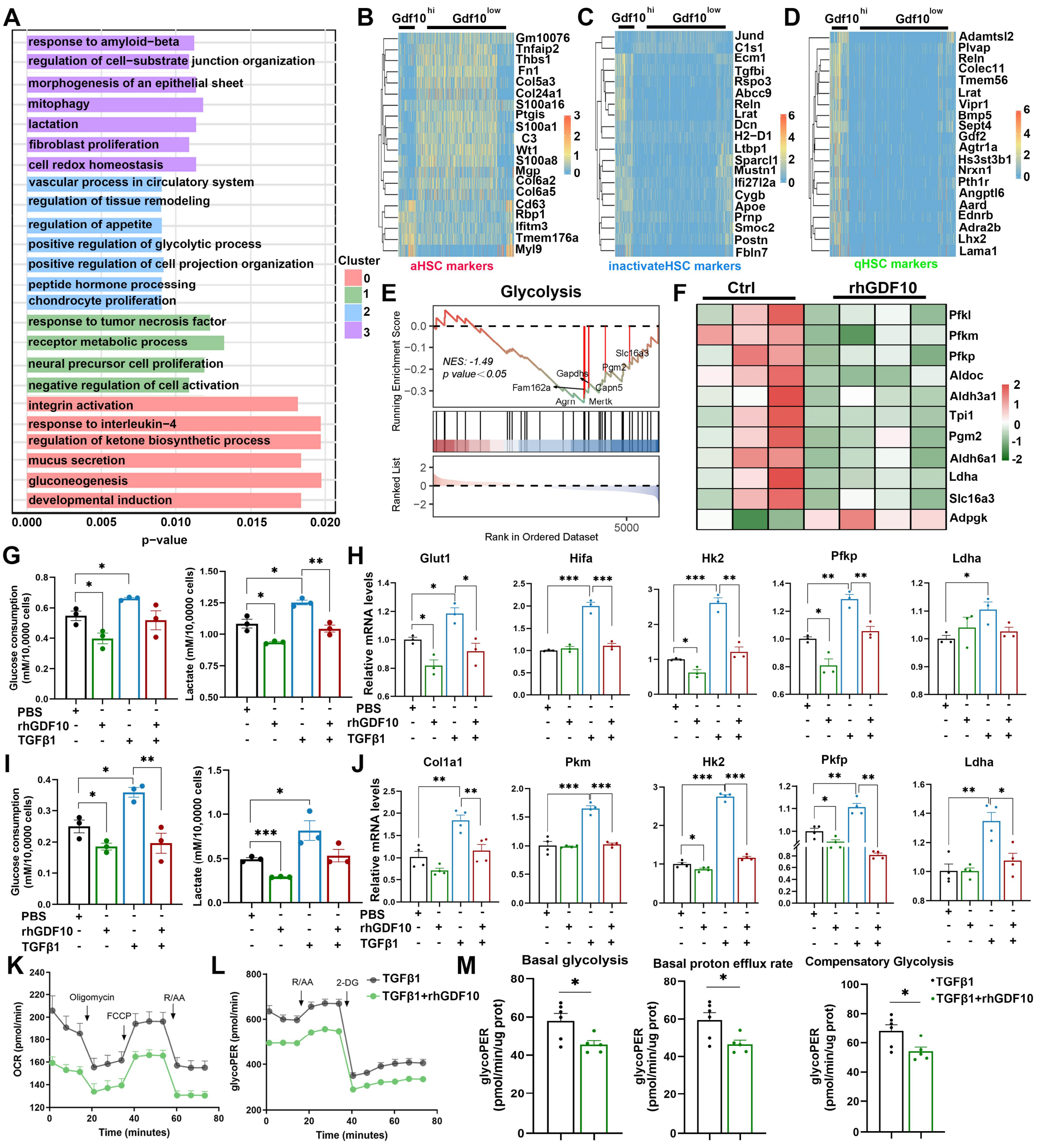

Figure S4. GDF10 functionally shifts activated HSCs into a quiescent state, related to figure 5. A. Representative GO terms enriched with DEGs in four subclusters of HSCs (CRA007803). B-D. Heatmap showing the aHSC, inactivate HSC and qHSC

marker genes of DEGs from *Gdf10^{low}* versus *Gdf10^{hi}* HSC. E. GSEA analysis of the pathway between Ctrl versus rhGDF10 treated immortalized HSCs. F. Heatmap showing the glycolysis pathway genes of DEGs from Ctrl or rhGDF10 treated immortalized HSCs. G. The concentration of glucose uptake and lactate secretion levels in the supernatant in Ctrl or rhGDF10 treated immortalized HSCs after culture for 24 h. H. qPCR analysis of glycolysis related genes in TGFβ1 or rhGDF10 treated immortalized HSCs. I. The concentration of glucose uptake and lactate secretion levels in the supernatant in Ctrl or rhGDF10 treated LX2 after culture for 24 h. J. qPCR analysis of glycolysis related genes in TGFβ1 or rhGDF10 treated LX2. K-M. OCR (K), GlycoPER (L) and qualification (M) in LX2 with rhGDF10 or TGFβ1 treatment in OCR or GlycoPER process, respectively. Data are representative of three independent experiments. Data are presented as mean ± SEM. *P < 0.05, **P < 0.01, ***P < 0.001.

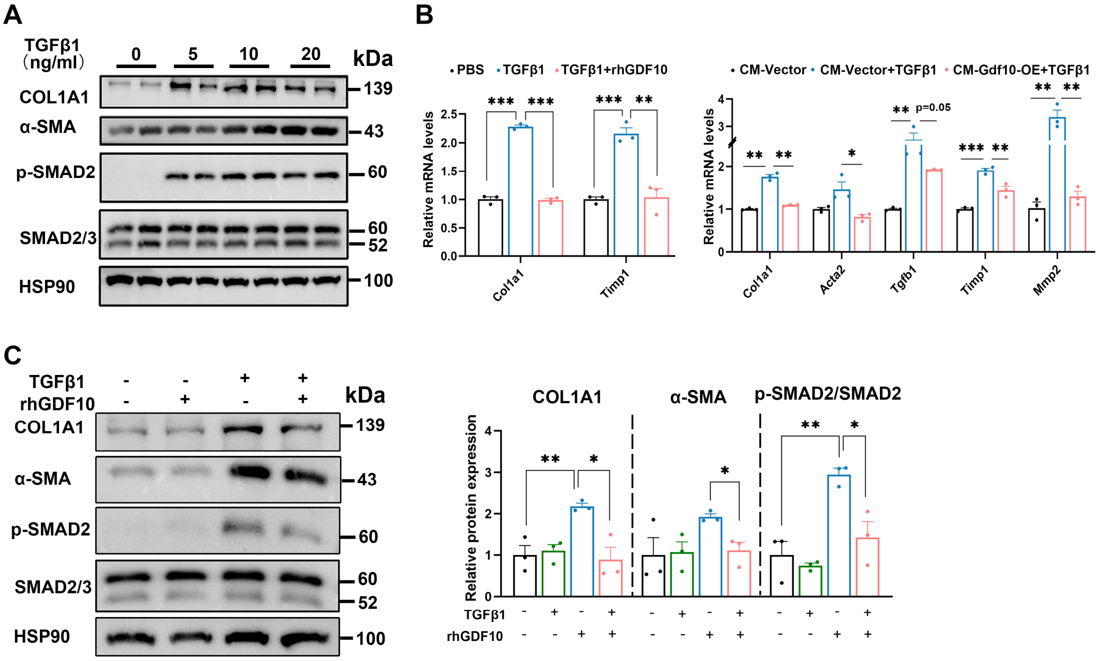

Figure S5. GDF10 exerts anti-fibrotic effects by competitively inhibiting TGF-β–dependent SMAD2/3 signaling in HSCs, related to figure 6. A. Immunoblot analysis of COL1A1, α-SMA and phosphorylation of SMAD2 of the HSCs with difference dose of TGFβ1 treatment. B, C. qPCR analysis of indicated genes in TGFβ1 or rhGDF10 (B), and TGFβ1 or CM-*Gdf10-OE* treated HSCs (C). D. Immunoblot analysis of COL1A1, α-SMA and phosphorylation of SMAD2 of the LX2 with TGFβ1 or

rhGDF10 treatment. Data are representative of three independent experiments and are presented as mean ± SEM. *P < 0.05, **P < 0.01, ***P < 0.001.

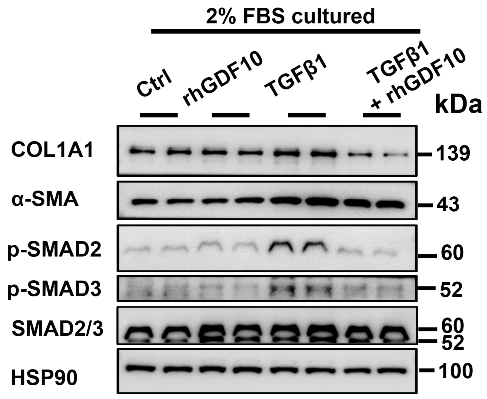

Figure S6. Immunoblot analysis of COL1A1, α-SMA and phosphorylation of SMAD2 and SMAD3 in HSCs cultured in 2% FBS treated with TGFβ1 or rhGDF10.

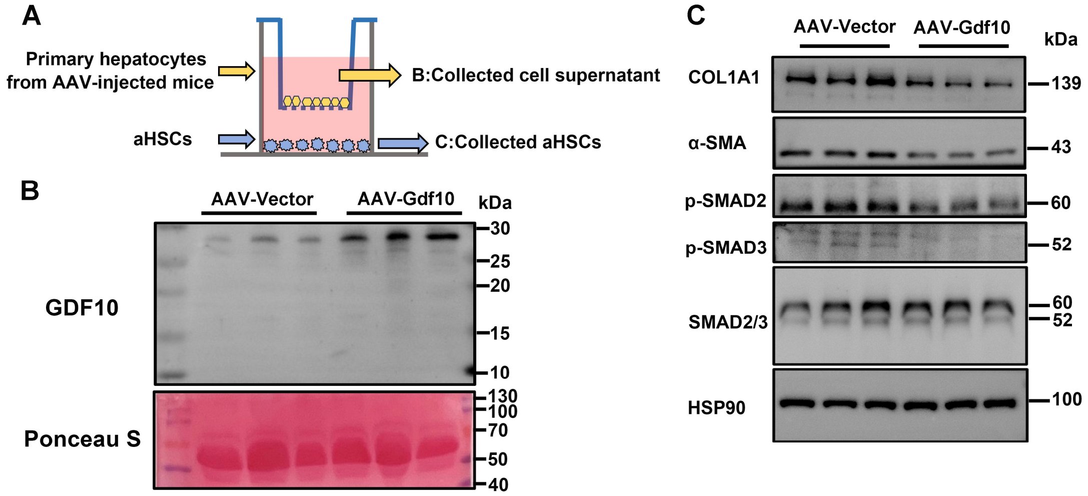

Figure S7. Analysis of hepatocytes-HSCs co-culture system. A. Schematic of co-cultured primary hepatocytes and aHSCs. B. Immunoblot analysis of GDF10 protein levels in the conditioned medium collected from primary hepatocytes. Ponceau S staining is shown as an internal loading control. C. Immunoblot analysis of COL1A1, α-SMA and phosphorylation of SMAD2 and SMAD3 in aHSCs. Data shown are representative of three independent experiments.

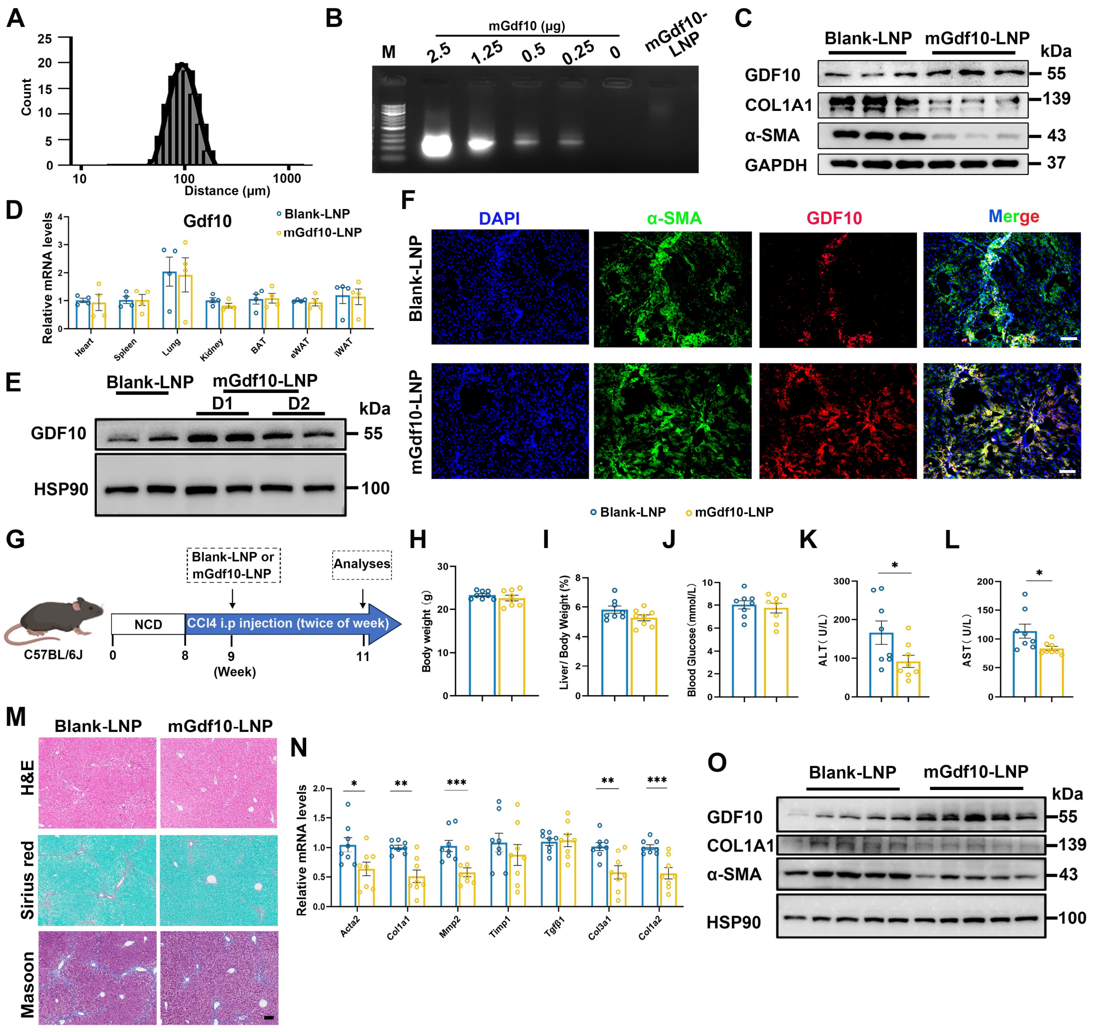

Figure S8. LNP-encapsulated *mGdf10* exhibits anti-fibrotic effects, related to figure 7. A. Nanoparticle tracking analysis of the *mGdf10*-LNP. B. mRNA with different total amount was analyzed by 1% agarose gel electrophoresis. 1.25 ug *mGdf10*-LNP was loaded into the last lane to detect encapsulation efficiency. C. Immunoblot analysis of COL1A1, α-SMA and phosphorylation of SMAD2 of the cultured aHSCs with LNP or *mGdf10*-LNP treatment. D, E. qPCR (D) and immunoblot (E) analysis of *Gdf10* mRNA levels of multiple tissues in LNP (n = 4) or *mGdf10*-LNP (n = 4) treated mice. F. IF analysis of GDF10 and α-SMA co-expression of the liver in LNP or *mGdf10*-LNP treated mice. scale bars, 100 μm. G. Experimental design for H-O. H-L. Measurement of body weight (H), liver/body weight (I), blood glucose levels (J), serum ALT (K) and AST (L) in the CCl4-induced fibrosis mice treated with *mGdf10*-LNP (n = 8 for control and n = 8 for *mGdf10*-LNP group). M. Representative images of H&E, Sirius Red, and Masson staining in the CCl4-induced fibrosis mice treated with *mGdf10*-LNP. scale bars, 50 μm. N, O. qPCR (N) and immunoblot (O)

analysis of indicated genes of the liver in the CCl4-induced fibrosis mice treated with *mGdf10*-LNP. Data are presented as mean ± SEM. *P < 0.05, **P < 0.01, ***P < 0.001.

Table S1. Primer sequences for qPCR of specific genes.

Gene	Species	Forward Primers	Reverse Primers
36b4	Mouse	GAAACTGCTGCCTCACATCCG	GCTGGCACAGTGACCTCACACG
Mmp13	Mouse	TGTTTGCAGAGCACTACTTGAA	CAGTCACCTCTAAGCCAAAGAAA
Timp1	Mouse	CGAGACCACCTTATACCAGCG	ATGACTGGGGTGTAGGCGTA
Mmp2	Mouse	CAACGGTCGGGAATACAGCAGC	TGGAAGCGGAACGGGAACTTG
Tgfb1	Mouse	ACCATGCCAACTTCTGTCTGGGAC	ACAACTGCTCCACCTTGGGCTTG
Acta2	Mouse	CTGACAGAGGCACCACTGAA	CATCTCCAGAGTCCAGCACA
Col3a1	Mouse	CTGTAACATGGAAACTGGGGAAA	CCATAGCTGAACTGAAAACCACC
Col1a2	Mouse	AGGTCCTAATGGAGATGCCG	CACAGGGCCTTCTTTACCAG
Col1a1	Mouse	AAGAGGCGAGAGAGGTTTCC	AGAACCATCAGCACCTTTGG
Gdf10	Mouse	GCAAGCCCCGAGCTAAGAA	GATTGAGGAGATGTCCTTGGC
Gfap	Mouse	GGGGCAAAAGCACCAAAGAAG	GGGACAACTTGTATTGTGAGCC
Bambi	Mouse	CATTGCTGGCGGACTGATCTT	CTTGCCCCTTCTTGGAATGGT
Insig1	Mouse	CTGTATTGCCGTGTTCGTTG	CTTCGGGAACGATCAAATGT
Pparg	Mouse	TATGGAGTGACATAGAGTGTGCT	GTCGCTACACCACTTCAATCC
Acox1	Mouse	GCCTGCTGTGTGGGTATGTCATT	GTCATGGGCGGGTGCAT
Ppara	Mouse	GCAGTGCCCTGAACATCGA	CGCCGAAAGAAGCCCTTAC
Acadm	Mouse	GCTGGAGACATTGCCAATCA	GGCGTCCCTCATCAGCTTCT
Hmgcs2	Mouse	GACATCAACTCCCTGTGCCTG	GATGTCAGTGTTGCCTGAATC
Cd36	Mouse	TTAGATGTGGAACCCATAACTGGA	TTGACCAATATGTTGACCTGCAG
Srebp1c	Mouse	CACCAGCATAGGCGAAGGA	ATGCCGACCAGATTCCCTAA
Fasn	Mouse	GGAGGTGGTGATAGCCGGTAT	TGGGTAATCCATAGAGCCCAG
Fsp27	Mouse	TCGACCTGTACAAGCTGAACCCT	AGGTGCCAAGCAGCATGTGACC
Hmgcs1	Mouse	AAAGATGTGGGAATCGTTGC	GGCCGATGGTATACTTTCCA
Pepck	Mouse	CTGCATAACGGTCTGGACTTC	CAGCAACTGCCCGTACTCC
Pgc1a	Mouse	TATGGAGTGACATAGAGTGTGCT	GTCGCTACACCACTTCAATCC
Fbp2	Mouse	GGTTCCATGGTGGCTGATGT	GGCCACAGGATTGCATTCAT
Trem2	Mouse	CAGCACCTCCAGGAATCAAGA	AGGATCTGAAGTTGGTGCCC
Ccl2	Mouse	AGGTCCCTGTCATGCTTCTG	TCTGGACCCATTCCTTCTTG
Gpnmb	Mouse	GAGCACAACCAATTACGTGGCT	GGTGATATTGGAACCCACCAGA
Tnfa	Mouse	AGCCCCCAGTCTGTATCCTT	CTCCCTTTGCAGAACTCAGG
Glut1	Mouse	CCTGTCTCTTCCTACCCAACC	GCAGGAGTGTCCGTGTCTTC
Hifa	Mouse	ACCTTCATCGGAAACTCCAAAG	CTGTTAGGCTGGGAAAAGTTAGG
Hk2	Mouse	CCGCCGTGGTGGACAAGATA	AGCAGTGATGAGAGCCGCTC
Pfkp	Mouse	CGCCTATCCGAAGTACCTGGA	CCCCGTGTAGATTCCCATGC
Ldha	Mouse	GGTGCATCCCATTTCCAC	GTCTGCGCTCTTCTTCAGG
36B4	Human	AGGCGTCCTCGTGGAAGTGA	GCGGATCTGCTGCATCTGCT

COL1A1	Human	AACATGACCAAAAACCAAAAGTG	CATTGTTTCCTGTGTCTTCTG
PKM	Human	ATGTCGAAGCCCCATAGTGAA	TGGGTGGTGAATCAATGTCCA
HK2	Human	GAGCCACCACTCACCCTACT	CCAGGCATTCGGCAATGTG
PFKP	Human	GCATGGGTATCTACGTGGGG	CTCTGCGATGTTTGAGCCTC
LDHA	Human	ATGGCAACTCTAAAGGATCAGC	CCAACCCCAACAACTGTAATCT
